# Extreme Kinetic Stability and RNase Resistance of Human Telomerase RNA G‐Quadruplexes Overcome by DHX36 Helicase

**DOI:** 10.1002/advs.202522779

**Published:** 2026-02-15

**Authors:** Qun Luo, Yashuo Zhang, Yingxian Qian, Huiying Zhan, Wenqiang Wu, Huijuan You

**Affiliations:** ^1^ Hubei Key Laboratory of Natural Medicinal Chemistry and Resource Evaluation School of Pharmacy, Tongji Medical College Huazhong University of Science and Technology Wuhan China; ^2^ Department of Pharmacy Ruijin Hospital Shanghai Jiao Tong University School of Medicine Shanghai China; ^3^ State Key Laboratory of Crop Stress Adaptation and Improvement Academy for Advanced Interdisciplinary Studies School of Life Sciences Henan University Kaifeng China

**Keywords:** DHX36, G‐quadruplex, helicase, RNA, RNase Resistance, single‐molecule

## Abstract

RNA G‐quadruplexes (G4s) formed at the 5′‐end of the RNA component of human telomerase (hTR) are known to directly affect telomerase activity. However, the unfolding kinetics of hTR_1–18_ G4s at physiological K^+^ conditions have not been analyzed due to their extremely high thermal stability (*T*_m_ > 80°C). Here, we overcome this challenge by measuring the unfolding rates of hTR_1–18_ RNA G4s using single‐molecule magnetic tweezers and bulk RNase digestion assays. We found that hTR_1–18_ RNA G4s exhibited exceptionally high mechanical stability and slow unfolding rates (10^−7^ s^−1^) in physiologically relevant 100 mm KCl buffer. Furthermore, we directly determined the digestion rate (*k*_dig_ = 1.2 × 10^−5^ s^−1^) of hTR_1–18_ RNA G4s in the presence of a high concentration of 1 U/µL RNase T1, which suggests that the RNase T1 can digest folded RNA G4 structure. Importantly, G4‐specific helicase DHX36 efficiently overcame this kinetic barrier, significantly reduced the fraction of folded hTR_1–18_ G4s from 99% to 16%, and rendered them susceptible to RNase degradation. These results illuminate the remarkable kinetic stability of RNA G4s and highlight the crucial role of helicase‐dependent unfolding in controlling the persistence of RNA G4s in cells.

## Graphical abstract

The RNA G‐quadruplexes formed at the 5'‐end of the RNA component of human telomerase possess extreme slow unfolding rate, rendering them resistant to nuclease degradation. The helicase DHX36 overcame this kinetic barrier and significantly reduced the folded fraction.

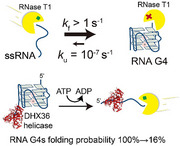

## 1 Introduction

Guanine‐rich RNAs can fold into four‐stranded secondary structures known as G‐quadruplexes(G4s). These structures are prevalent across various RNA species, including mRNA, microRNA, and long non‐coding RNA (lncRNA), and are implicated in diverse biological processes [1, 2, 3], such as translation regulation [4, 5], mRNA splicing [6], enzymatic activity modulation [7], and telomere maintenance [8, 9]. Live‐cell imaging studies have provided compelling evidence supporting the existence and dynamic behavior of RNA G4 structures within living cells [10, 11, 12, 13, 14]. Transcriptome‐wide methods, including reverse transcriptase‐stalling (rG4‐seq) [15, 16], G4‐specific small molecules binding (TASQs [17] and Colistin [18]), have been developed to map the distribution of RNA G4s. However, previous studies using dimethyl sulfate methylates (DMS) footprinting and RNase stop assay suggested that RNA G4s are globally unfolded in eukaryotic cells, highlighting the dynamic folding/unfolding nature of G4s in vivo [16]. Therefore, characterizing the biophysical properties, such as folding/unfolding rates of G4s and their regulations, is important for understanding their lifetime and function in vivo [19].

Among the biologically significant RNA G4s, the structure formed within the RNA subunit of human telomerase (hTR) is of particular interest. Telomerase, the ribonucleoprotein complex responsible for synthesizing telomeric DNA, consists of the telomerase reverse transcriptase (TERT) and the telomerase RNA (TR) component [20, 21]. The 5′‐end of hTR contains a guanine‐rich sequence that can form G4 structures, which interfere with the formation of the P1 helix and thus affect the template boundary for telomere reverse transcription [22, 23]. Specifically, the G4 structure formed by the 18 nucleotides at the 5′‐end of hTR (hTR_1–18_) has been detected from total cellular RNA using reverse transcriptase stalling assay [24] and solved by NMR spectroscopy [25], supporting the formation of a parallel‐stranded G4 with a bulge. This hTR_1–18_ G4s can mediate the binding of the DEAH‐box helicase DHX36 (also known as RHAU helicase) to telomerase, a key factor that influences telomerase activity [23, 26].

RNA G4s are generally known to possess superior thermodynamic stability and faster folding kinetics when compared with their DNA counterparts [27, 28, 29, 30, 31]. For example, stopped‐flow measurements have shown that telomeric repeat‐containing RNA (TERRA) G4s in 90 mm KCl (10 mm Tris (pH 7.0)) complete folding within 400 ms [32]. The hTR_1–18_ RNA G4s themselves are highly stable, exhibiting a melting temperature (*T*_m_) higher than 80°C in a physiologically relevant 100 mm KCl buffer. This high thermal stability makes determining their unfolding kinetics challenging, but it is nonetheless essential for understanding the structure formation and persistence (lifetime) in vivo. Although RNase T1 digestion experiments suggest a long lifetime for several folded RNA G4s (TERRA and a 5′‐UTR RNA), based on short‐term resistance to RNase T1 digestion for 5 min at 150 mm salt concentration [33, 34], comprehensive longer‐term stability assessments are lacking. Furthermore, the coexistence of multiple RNA G4s structural conformations in solution and the limitations of traditional ensemble average methods impede the quantitative analysis of individual G4 structures. Therefore, a quantitative approach is required to fully resolve the unfolding dynamics and extraordinary stability of the hTR_1–18_ RNA G4s.

Single‐molecule approaches enable direct measurements of the folding/unfolding dynamics of G4s and facilitate the discovery of diverse G4 topologies [35, 36, 37, 38, 39]. Especially, single‐molecule force spectroscopies such as optical tweezers, magnetic tweezers, and atomic force microscopy have provided important information on the mechanical stability and folding/unfolding kinetics of G4 structures [36, 37]. While the mechanical stability and folding/unfolding kinetics of parallel‐stranded DNA G4s have been systematically studied [40, 41], data on RNA G4s remain sparse. Previous optical tweezers measurements on RNA G4s, including TERRA G4s [42, 43] and hTR_10‐27_ sequence [44], reported unfolding force generally below 40 pN, which is typically lower than those observed for parallel‐DNA G4s [41]. However, the functional lifetime of G4s in vivo is closely related to their kinetic stability (unfolding rate); therefore, investigating the unfolding rates of highly stable RNA G4s (such as those with *T*_m_ > 80°C) remains necessary. Crucially, the unfolding rates of hTR_1–18_ RNA G4s under physiological K^+^ buffer remain poorly understood, which is essential for a complete understanding of its dynamics and role in telomerase.

The high stability of RNA G4s also poses a regulatory challenge. The helicase DHX36 is known to interact with telomerase RNA [23, 26, 45] and serves as the major G4 resolvase in HeLa cells [46]. Loss of DHX36 activity leads to an accumulation of translationally inactive target mRNAs with RNA G4 structures in untranslated regions [47]. The crystal structure of DHX36 and single‐molecule FRET analysis have provided the molecular basis for DHX36's recognition and unfolding of G4s [48, 49, 50], and magnetic tweezers have analyzed DHX36 destabilization of DNA G4s [51]. However, the effects of DHX36 on the mechanical stability of RNA G4s have not been addressed by force‐based measurements. Elucidating this mechanism is essential for understanding how helicases resolve these robust RNA G4 structures to ensure proper biological function.

**FIGURE 1:**
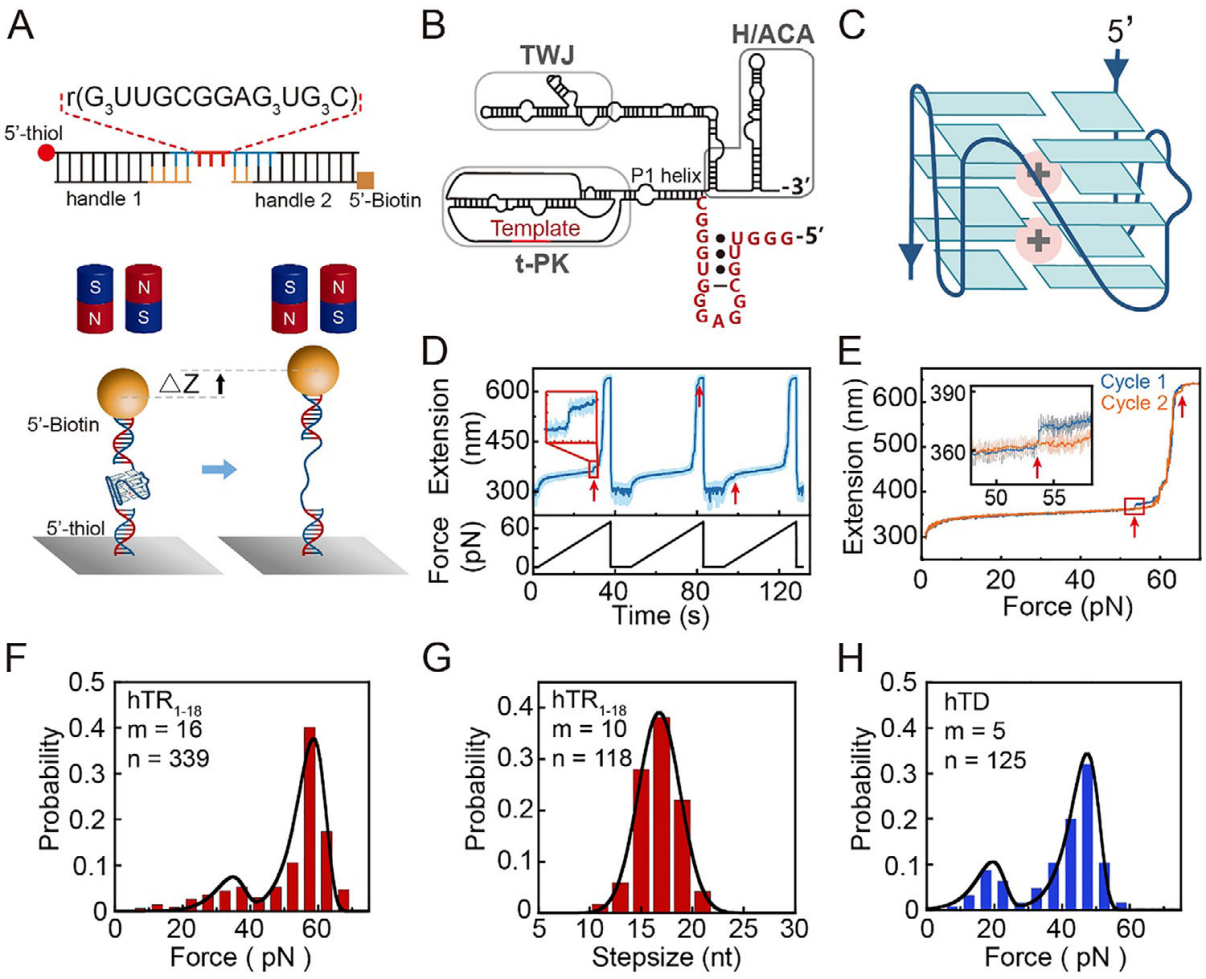
Single‐molecule magnetic tweezers analysis of the mechanical stability of hTR_1–18_ RNA G4s. (A) Schematic illustration of the experimental setup. The hTR_1–18_ RNA G4‐forming sequence (rGGGUUGCGGAGGGUGGGC) is flanked by two double‐stranded DNA handles and tethered between a coverslip and a paramagnetic bead. (B) Proposed secondary structure of the full human telomerase RNA [21]. (C) Proposed folding topology of hTR_1–18_ RNA G4 in K^+^ solution [25]. (D) Representative force‐ramp stretching cycles. The G4 unfolding events are marked by an abrupt increase in extension jump (red arrow). (E) Overlay of force‐extension curve from the first (cycle 1) and second (cycle 2) stretching cycles shown in (D). The inset is an enlarged view of the red rectangle. (F) Unfolding force distribution for the hTR_1–18_ RNA G4s. Data were collected in a 100 mm KCl buffer and fitted with Bell's model to determine the unfolding rate. (G) Unfolding step sizes of the hTR_1–18_ RNA G4s. Data were fitted with the Gaussian equation. (H) Unfolding force distribution of hTD G4s. Data were obtained in a 100 mm KCl buffer and fitted with Bell's model. Data were compiled from n events across m molecules.

Here, we combine single‐molecule magnetic tweezers with bulk RNase digestion assays to fill these critical gaps. We first investigated the mechanical stability of the hTR_1–18_ RNA G4s (Figure 1A–C). We demonstrate that the hTR_1–18_ RNA sequence can form multiple G4 structures, with the predominant conformation exhibiting extremely high mechanical stability and slow unfolding rate. We analyzed this mechanical stability across different K^+^ concentrations to determine the role of the K^+^ in the kinetic stability. Given that this kinetic stability likely confers resistance to RNase degradation, we further measured the RNase T1 and RNase A digestion rate of hTR_1–18_ RNA G4s to determine the functional implication of their long lifetime. Finally, we analyzed the effects of G4‐specific helicase DHX36 on the kinetic stability and RNase resistance of the hTR_1–18_ RNA G4s. These quantitative measurements provide insights into the intrinsic kinetic stability of hTR_1–18_ RNA G4s and their biological implications for G4 function and regulation.

## 2 Results

### 2.1 hTR_1–18_ RNA G4s Exhibit Extremely High Mechanical Stability and Slow Unfolding Rates in Physiologically Relevant Concentrations of KCl

Figure 1A shows the experimental setup using magnetic tweezers. A single‐stranded DNA‐RNA‐DNA chimeric oligonucleotide containing the hTR_1–18_ RNA sequence was synthesized and ligated to two double‐stranded DNA (dsDNA) handles and tethered between a coverslip and a paramagnetic bead (Figure S1). Figure 1D shows representative repeating stretching cycles of the hTR_1–18_ RNA in the presence of 100 mm KCl and 10 mm Tris‐HCl (pH 8.0) buffer. The force‐ramp procedure involved linearly increasing the force from 1 to 70 pN at a constant loading rate of 2 pN/s. Each stretching cycle was followed by a rapid force reduction back to 1 pN, where the construct was held for a certain duration to allow the hTR_1–18_ RNA G4s to refold. The G4 unfolding transition was detected as a sudden extension jump of 7–8 nm (Figure 1D,E, red arrows). We noted that the dsDNA handles exhibited a significant elongation (approximately 200 nm, or 0.7 times the counter length) at a force near 65 pN, characteristic of the B‐form DNA to S‐form DNA transition [52]. This large fluctuation in dsDNA extension made it challenging to detect the G4 unfolding signal at forces greater than 60 pN. However, by overlaying multiple force‐extension curves and comparing the stretching vs relaxing curves, we were able to detect the hTR_1–18_ G4 unfolding events even in the high‐force region (Figure 1E and Figure S2). Notably, while previously analyzed parallel‐stranded DNA G4s typically unfold below 60 pN (>95% of events) [40, 41], a large fraction of hTR_1–18_ RNA G4s remained folded even when the force reached 60 pN, demonstrating their significantly higher mechanical stability (Figure 1D).

To determine the unfolding rates of RNA G4s, we measured the unfolding force distribution of hTR_1–18_ RNA G4s from 339 unfolding events observed across 16 individual molecules in 100 mm KCl buffer (Figure 1F). The distribution showed two distinct peaks for hTR_1–18_ G4s, indicating the presence of two folded conformations with differing stabilities. The predominant population (84% of events) unfolded at a high force of 58 ± 4 pN (average ± standard deviation), while a minor population (16% of events) unfolded at 36 ± 4 pN (Figure 1F). Analysis of long trajectories from individual molecules confirmed that a single molecule could stochastically exhibit unfolding events corresponding to both the high‐force and low‐force peaks (Figure S3). Fitting these data to Bell's model [53] using transition distance Δ*x* = 1 nm yielded the corresponding zero‐force unfolding rate $k_{u}^{0}$ of (3.1 ± 0.3) × 10^−7^ s^−1^ and (1.1 ± 0.5) × 10^−4^ s^−1^ (average ± fitting error), respectively. The exceptionally slow $k_{u}^{0}$ for the dominant species indicates an extreme kinetic stability. The unfolding step size for hTR_1–18_ G4s was 17 ± 2 nt (Figure 1G), consistent with the number of nucleotides involved in a fully‐folded hTR_1–18_ G4 structure. These findings confirm that hTR_1–18_ sequences form fully‐folded G4s, with the dominant species exhibiting remarkably high mechanical stability and slow unfolding rate. Furthermore, the hTR‐nobulge variant (lacking the internal cytosine bulge) exhibited mechanical stability comparable to that of wild‐type hTR_1–18_ (Figure S4), suggesting that this single nucleotide cytosine bulge does not significantly affect the mechanical stability.

For comparison, we analyzed the stability of the DNA counterpart to hTR_1–18_ RNA, hTD (Table S1). The hTD also forms a parallel‐stranded G4 structure in 100 mm KCl buffer, as indicated by the CD spectrum (Figure S5). The unfolding force distribution of hTD revealed two distinct peaks: 46 ± 5 pN (77%) and 19 ± 5 pN (23%), with the corresponding $k_{u}^{0}$ determined to be (4.8 ± 0.3) × 10^−6^ s^−1^and (4.3 ± 0.8) × 10^−3^ s^−1^ (average ± fitting error) (Figure 1H). These results demonstrate that RNA G4s exhibit significantly greater mechanical stability compared to their DNA counterparts, with unfolding rates approximately 10‐fold slower.

### 2.2 Mechanical Stability and Unfolding Rates of hTR_1–18_ RNA G4s are Largely Independent of K^+^ Concentration Above 10 mm

**FIGURE 2:**
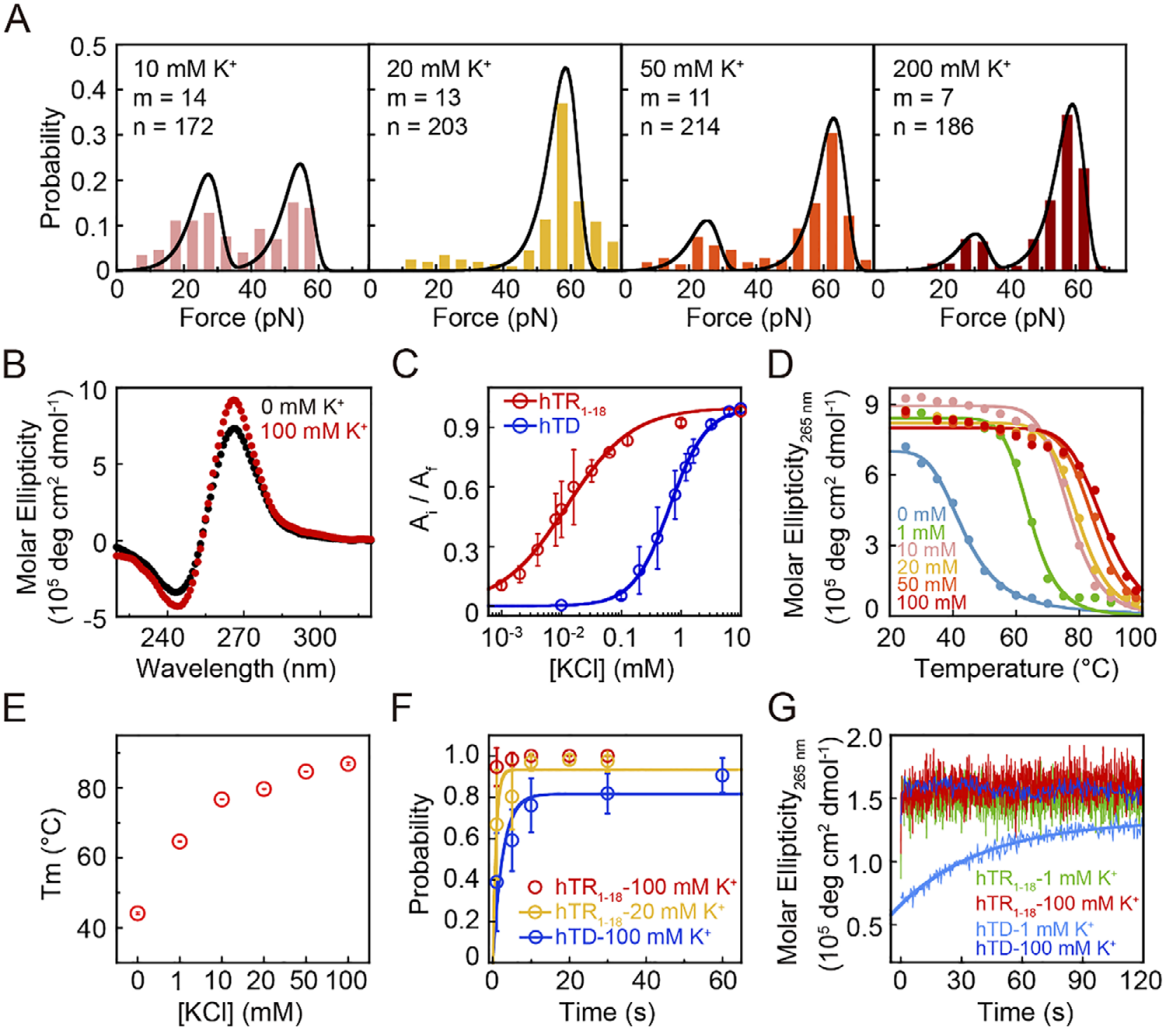
K^+^ concentration‐dependent unfolding and folding rates of hTR_1–18_ RNA G4s. (A) Unfolding force distributions of hTR_1–18_ G4s. Data were obtained in 10 mm KCl (pink), 20 mm KCl (yellow), 50 mm KCl (orange), and 200 mm KCl (red) buffer. Data were compiled from n events across m molecules. Solid lines are fits to the Bell's model. (B) CD spectra of hTR_1–18_ measured in the presence of 0 (black) or 100 (red) mm K^+^. (C) K^+^ titration curves of hTR_1–18_ RNA G4s (red circle) and hTD DNA G4s (blue circle). Data were extracted from CD spectra at 265 nm. Solid lines are fits to the Hill equation. (D) CD melting curves of hTR_1–18_ G4s measured in different concentrations of K^+^. Data were measured at 265 nm and fitted by a sigmoid function. (E) Melting temperatures (*T*_m_) of hTR_1–18_ at varying K^+^ concentrations (0–100 mm). (F) The time evolution of the folding probability *p*_fold_(*t*) of hTR_1–18_ G4s and hTD G4s. Open circles indicate experimental *p*_fold_(*t*) measured based on more than 80 refolding/unfolding cycles from at least three molecules. The solid lines with corresponding colors are single‐exponential fits. (G) CD Stopped‐flow kinetics traces for folding reactions after mixing the G4‐forming oligonucleotides with K^+^. Error bars in (C), (E), and (F) represent the standard deviation from at least three independent experiments.

Given that K^+^ is essential for coordinating the G‐tetrads and stabilizing the G4 core, we next sought to determine whether the kinetic stability of the hTR_1–18_ RNA G4s is dependent on the cation concentration. We quantified the unfolding force distribution of hTR_1–18_ RNA G4s across a range of 10–200 mm KCl using magnetic tweezers. The unfolding force distributions of hTR_1–18_ RNA G4s consistently showed a major population (> 75% of the events) with unfolding force peaks observed at 56 ± 9 pN (10 mm KCl), 58 ± 4 pN (20 mm KCl), 61 ± 5 pN (50 mm KCl), and 58 ± 5 pN (200 mm KCl), respectively (Figure 2A). Their zero‐force unfolding rates determined by fitting the data with Bell's model were in the order of 10^−7^ s^−1^ (Table 1). These results suggest that the mechanical stability and unfolding rates of hTR_1–18_ G4s are insensitive to K^+^ concentration changes within the 20–200 mm range. We observed a notable change only at the lowest concentration of 10 mm KCl tested. At this concentration, the stable conformation (unfolding force peak at 56 ± 9 pN) becomes less dominant, occupying only 53% of the population, with the remaining 47% population unfolding at a much lower force of 23 ± 9 pN. This suggests that extreme stability of hTR_1–18_ G4s is compromised when the K^+^ concentration falls below a critical threshold of ∼10 mm, likely where K^+^ coordination becomes insufficient. The fit to the Bell's model at 10 mm KCl is less satisfactory, which we attribute to increased structural heterogeneity. The possible coexistence of partially folded intermediates and alternative topologies at this critical K^+^ concentration may cause the kinetics to deviate from standard Bell's model assumptions. We were unable to determine the mechanical stability of G4s at KCl concentrations below 10 mm due to the reduced stability of the dsDNA handles in low‐salt conditions, leading to breakage of the DNA tethers.

**TABLE 1:** Unfolding force, zero‐force unfolding rate ($k_{u}^{0}$), and steady‐state folding probability (*p*_st_) of hTR_1–18_ G4s and hTD G4s.

Name	(KCl) (mm)	Unfolding force (pN)	$k_{u}^{0}$ (s^−1^)
hTR_1–18_ RNA G4s	10	23 ± 9 (47%) 56 ± 9 (53%)	(6.5 ± 2.0) × 10^−4^ (8.5 ± 2.1) × 10^−7^
	20	58 ± 4	(3.2 ± 0.4) × 10^−7^
	50	25 ± 4 (25%) 61 ± 5 (75%)	(1.0 ± 0.3) × 10^−3^ (1.08 ± 0.09) × 10^−7^
	100	36 ± 4 (16%) 58 ± 4 (84%)	(1.1 ± 0.5) × 10^−4^ (3.1 ± 0.3) × 10^−7^
	200	30 ± 4 (18%) 58 ± 5 (82%)	(3.1 ± 0.4) × 10^−4^ (2.81 ± 0.06) × 10^−7^
hTD DNA G4s	100	19 ± 5 (23%) 46 ± 5 (77%)	(4.3 ± 0.8) × 10^−3^ (4.8 ± 0.3) × 10^−6^

To determine the threshold concentration required for hTR_1–18_ G4s formation, we performed K^+^ titration experiments monitored by CD spectroscopy. Figure 2B shows the CD spectra of hTR_1–18_ in the absence and presence of 100 mm KCl. The characteristic spectra with a minimum at ∼240 nm and a maximum at ∼265 nm confirmed the formation of parallel‐stranded G4 structures. Notably, the hTR_1–18_ sequence forms a parallel‐stranded G4 structure even at near‐zero K^+^ concentrations, unlike its DNA counterpart (hTD), which requires K^+^ (Figure S6). This observation suggests RNA G4s folding may proceed through a collapsed, molten globule‐like state that does not strictly depend on high K^+^ concentration. As the K^+^ concentration increased from 0.001–10 mm, the CD spectra at ∼265 nm increased for both hTR_1–18_ and hTD sequences, indicating the progression of G4 folding (Figure S6). By fitting the CD signal change at 265 nm to the Hill equation, we determined the midpoint of K^+^ induced folding (*K*_0.5_) for hTR_1–18_ G4s to be 30 ± 8 µm and the Hill coefficient of *n*_H_ = 0.79 ± 0.06 (average ± fitting error) (Figure 2C). In contrast, the hTD DNA G4s required a much higher K^+^ concentration for folding, with a *K*_0.5_ = 542 ± 16 µm (*n*_H_ = 1.41 ± 0.04). The hTR_1–18_ G4s achieve complete folding at approximately 1 mm KCl, supporting the conclusion that concentrations above this level are sufficient for saturated RNA G4 formation. These results demonstrate that the hTR_1–18_ RNA G4s are more sensitive to K^+^ than their DNA counterpart, providing the structural basis for their high folding efficiency and stability.

We further assessed the hTR_1–18_ G4s’ thermal stability by measuring their *T*_m_ across a range of K^+^ concentration using CD melting experiments (Figure 2D and Figure S7). The *T*_m_ of hTR_1–18_ G4s showed a strong dependence on K^+^ concentration, increasing from 44.2°C ± 0.4°C in the absence of K^+^ to an exceptionally high 87.1°C ± 0.5°C in 100 mm K^+^, demonstrating a substantial enhancement in thermal stability (Figure 2E and Table S2). Thermodynamic parameters (Δ*H*, Δ*S*, and Δ*G*_25_) derived from van't Hoff analysis [54] further supported this observation (Table S2). Crucially, the most significant increases in both *T_m_* and ∆*G*_25_ occurred between 0 mm and 10 mm K^+^, after which the stability began to plateau. This correlates with the findings from our mechanical unfolding experiments, identifying ∼10 mm K^+^ as a threshold for the formation of extremely stable G4s. The *T*_m_ of hTD G4s in the presence of 100 mm K^+^ was measured for comparison, showing a *T*_m_ 22.5°C lower than that of hTR_1–18_ G4s, supporting that RNA G4s are thermodynamically more stable than their DNA counterparts (Figure S8). Taken together, our single‐molecule and CD titration experiments identified a critical threshold of approximately 10 mm KCl for the extremely stable G4 formation. In this regime (>10 mm K^+^), its thermal stability plateaus at a very high level, and its structure is maintained by exceptionally slow unfolding kinetics that are largely independent of further increases in K^+^ concentration.

### 2.3 Extreme Fast Folding Rates of hTR_1–18_ RNA G4s Revealed by Single‐Molecule and Stopped‐Flow Assay

In addition to the unfolding rate, the folding rate from ssDNA to the G4 structure is also important for G4s’ function in vivo. We measured time‐evolution folding probability *p*_fold_(*t*) of hTR_1–18_ RNA G4s using magnetic tweezers. The nucleic acid constructs were held at low force (1 pN) for various time intervals to permit G4 formation, followed by a force‐ramp stretch to determine if G4 folding had occurred during the holding time *t*. By repeating this stretching cycle *M* times, the folding probability was calculated as *p*_fold_ (*t*) = *N*/*M*, where *N* is the number of successfully folding events. The data was fitted by a single‐exponential equation *p*_fold_ (*t*) = *p*_st_ [1‐exp(‐*k*_fold_*t)] to determine the steady‐state folding probability (*p*_st_) and the apparent folding rate (*k*_fold_). The folding rate of hTR_1–18_ RNA G4s was too fast to be accurately measured under physiologically relevant K^+^ conditions, as it reached full folding within 1 s under 100 mm K^+^ conditions, thereby exceeding the time resolution limit of our magnetic tweezers. Consequently, we determined the folding rate at 20 mm K^+^. The hTR_1–18_ RNA G4s exhibited a high steady‐state folding probability (*p*_st_ = 95 ± 3%) and a fast apparent folding rate (*k*_fold_ = 1.5 ± 0.3 s^−1^) in 20 mm K^+^ (Figure 2F). This rate confirms that hTR_1–18_ RNA G4 folds significantly faster than its DNA counterpart hTD, which only achieves *p*_st_ = 82% ± 7% and a slower rate of *k*_fold_ = 0.4 ± 0.2 s^−1^ even in the presence of 100 mm K^+^.

To further quantify the rapid folding dynamics, we conducted CD stopped‐flow experiments. The oligonucleotides were rapidly mixed with K^+^ buffer, and the folding process was monitored by the change in the CD signal at 265 nm (Figure 2G). For the hTD DNA sequence in 1 mm K^+^, the kinetic trace fits well to a single exponential function, yielding a rate constant *k*_fold_ = 0.025 ± 0.004 s^−1^. However, the folding of hTD DNA in 100 mm K^+^ was completed within the 500‐ms dead time of the instrument, indicating a folding rate beyond our detection limit. The folding of hTR_1–18_ RNA G4s at both 1 mm and 100 mm K^+^ was too fast to resolve using our stopped‐flow instrument, firmly demonstrating that hTR_1–18_ RNA G4s fold faster than their DNA counterparts. We note that the folding rates measured via magnetic tweezers are typically slower than those of short oligonucleotides in solution. This discrepancy is attributed to the hydrodynamic drag caused by the large magnetic bead (2.8 µm in diameter) and the long DNA handles used in single‐molecule setups [55]. Collectively, both magnetic tweezers and stopped flow measurements suggest the extremely fast folding rate of RNA G4s relative to DNA G4s, consistent with a previous study on other G4‐forming RNA sequences like TERRA [32].

### 2.4 Digestion Rates of hTR_1–18_ RNA in the Presence of High Concentrations of RNase T1 and RNase A

**FIGURE 3:**
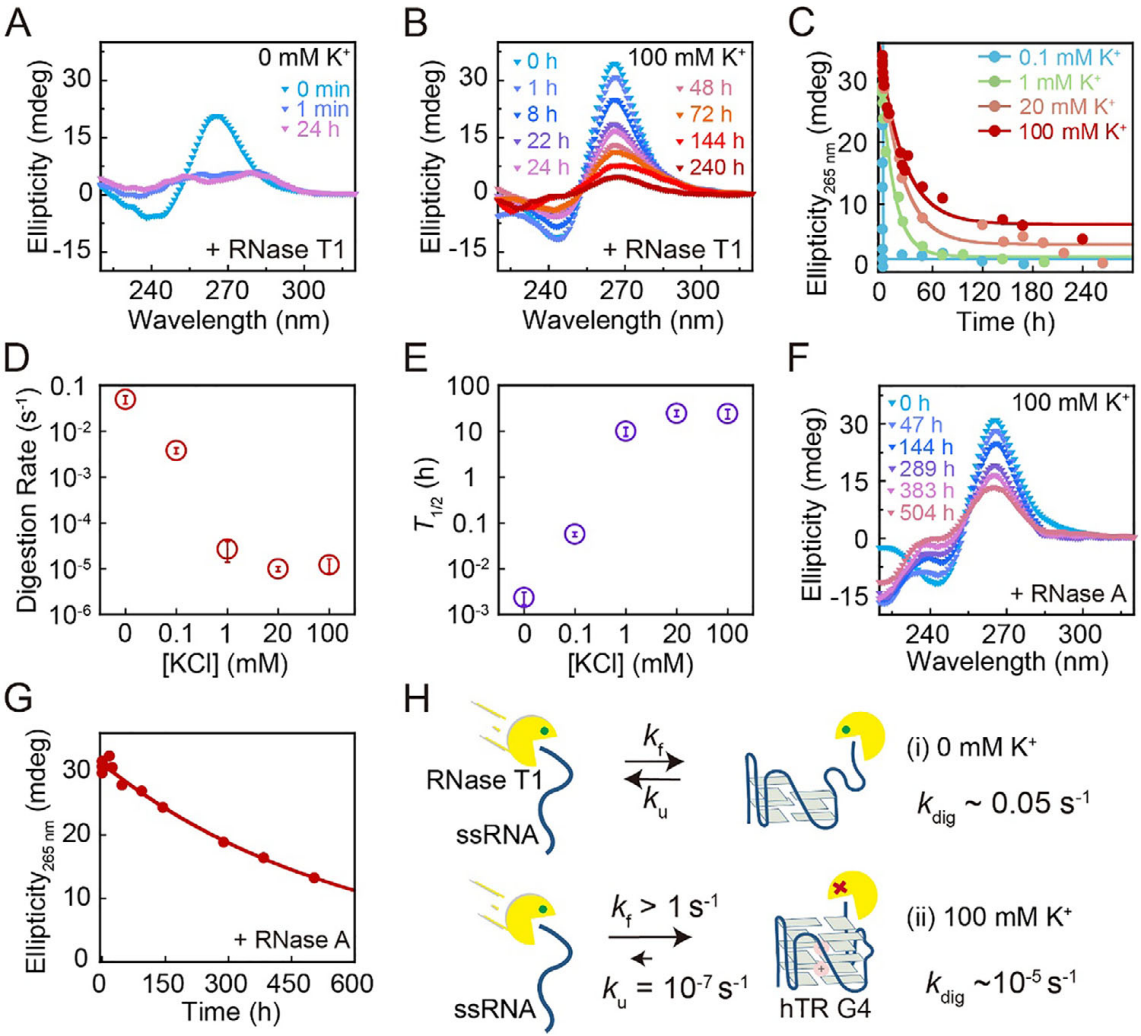
Digestion rates of hTR_1–18_ by RNase T1 and RNase A. (A–B) Time‐dependent CD spectra of hTR_1–18_ in 0 mm KCl buffer (A) or 100 mm KCl buffer (B) after the addition of 1 U/µL RNase T1. (C) Time evolution of CD signals at 265 nm for hTR_1–18_ treated with RNase T1 under varying K^+^ concentrations (0‐100 mm). The CD intensity changes reflect the extent of digestion of RNA. The data were fitted using an exponential decay function. (D) The digestion rate of hTR_1–18_ by RNase T1 derived from fitting the data in (C). (E) The half‐life (*T*_1/2_) of hTR_1–18_ in the presence of RNase T1 and varying K^+^ concentrations (0‐100 mm). (F) Time‐dependent CD spectrum of hTR_1–18_ in 100 mm KCl buffer after addition of 200 ng/µL RNase A. (G) Time evolution of CD signals at 265 nm for hTR_1–18_ digested by RNase A under 100 mm K^+^ conditions. (H) Schematic illustration of the K^+^‐dependent structural stability and RNase T1 resistance of the hTR_1–18_ G4s. At K^+^ concentrations below 0.1 mm, hTR_1–18_ forms a less stable G4 structure that is readily digested by RNase T1, whereas at 100 mm K^+^, it adopts a highly stable G4 structure resistant to enzymatic cleavage.

Given that the extreme kinetic stability likely affects the lifetime and persistence in vivo, we employed an RNase digestion assay to investigate the functional implications. Previous studies have shown that G4 structure can protect telomeric‐repeats‐containing RNA from RNase T1 digestion during short‐time (5 min) digestion [34]. However, the digestion rates of RNA G4s have not been quantified before. It remains unclear whether enzymes like RNase T1, which specifically cleaves RNA at single‐stranded guanosine residues, can digest folded RNA G4s or must act on the transiently unfolded single‐stranded RNA (ssRNA). To access the stability of hTR_1–18_ RNA G4s in the presence of RNase T1, we used CD spectroscopy to directly measure the digestion rates of pre‐folded hTR_1–18_ G4s by high concentrations of RNase T1 (1 U/µL). In a control experiment without K^+^, the unfolded RNA was rapidly degraded, with over 90% of the CD signal at 265 nm eliminated within 1 min (Figure 3A and Figure S9). The change in ellipticity of hTR_1–18_ at 265 nm was fitted with a single‐exponential function, yielding a degradation rate *k*_dig_ of hTR_1–18_ by RNase T1 of 0.05 ± 0.01 s^−1^ in the absence of K^+^. In contrast, in the presence of 100 mm K^+^, the folded G4s demonstrated extraordinary stability. The characteristic CD signal for parallel‐stranded G4s was retained for over a week, indicating extreme resistance to RNase T1 (Figure 3B). This enzymatic resistance is consistent with the slow unfolding rate of the G4 in 100 mm K^+^ conditions. By monitoring the CD signal decay at 265 nm over 240 h and fitting the data using a single‐exponential function (Figure 3C), we determined the digestion rates of hTR_1–18_ *k*_dig_ to be (1.2 ± 0.4) × 10^−5^ s^−1^, corresponding to a half‐life (*T*_1/2_) of 25 ± 6 h (Figure 3D,E). Crucially, this slow digestion rate is still faster than the unfolding rate of hTR_1–18_ G4s determined by magnetic tweezers experiments (Table 1). This finding suggests that RNase T1 can still directly digest the fully folded G4 structure, albeit at a slow rate.

We next investigated the effect of K^+^ concentration on the RNase T1 digestion rates of hTR_1–18_ RNA (Figure 3C and Figure S10). By fitting the CD signal decay, we obtained the digestion rates of (1.0 ± 0.1) ×10^−5^ s^−1^, (3 ± 1) × 10^−5^ s^−1^, and (3.8 ± 0.6) × 10^−3^ s^−1^ at 20, 1, and 0.1 mm KCl, respectively (Figure 3C,D). The hTR_1–18_ RNA maintained strong resistance to RNase T1 at K^+^ concentrations down to 1 mm. This observation is consistent with the slow unfolding rates and high thermal stability (*T*_m_) measured under these conditions (Figure 2). The digestion rate at 1 mm K^+^ was only increased ∼2‐fold faster than that at 100 mm K^+^. In contrast, decreasing the K^+^ concentration to 0.1 mm causes a dramatic, ∼300‐fold increase in digestion rate compared with 100 mm K^+^. This sharp acceleration correlates well with the significant decrease in the G4's melting temperature observed in a low K^+^ buffer (Figure 2 and Figure S7). This result suggests that the accelerated digestion rate at 0.1 mm K^+^ is due to the accelerated unfolding of hTR_1–18_ RNA G4s. Remarkably, even at this low K^+^ concentration (0.1 mM), the G4 structure still provides substantial protection. The digestion rate is approximately 13‐fold slower than that of unfolded RNA in the absence of K^+^ (*k*_dig_ = (0.05 ± 0.01 s^−1^). This indicates that even a less‐stable folded conformation can protect the RNA from RNase T1 digestion.

The digestion rate of RNase A (200 ng/µL) of hTR_1–18_ RNA was also analyzed, which preferentially cleaves RNA at pyrimidine residues (cytosine and uracil), found in the loop region of hTR_1–18_ RNA G4s. The results show that under 100 mm K^+^ condition, hTR_1–18_ RNA G4s exhibits strong resistance to RNase A digestion, which does not undergo complete degradation even after ∼500 h (Figure 3F,G). The digestion rate of hTR_1–18_ RNA G4s was *k*_dig_ = (5 ± 2) × 10^−7^ s^−1^ (half‐life of 380 ± 28 h) which is comparable to the intrinsic unfolding rates of hTR_1–18_ RNA G4s (10^−7^ s^−1^), suggesting that the digestion of hTR_1–18_ RNA G4s by RNase A require the unfolding of G4 structure and expose the loop region to RNase A. This observation suggests that the G4 loop or bulge region is relatively protected from RNase A cleavage. Our quantitative analysis of unfolding rates and digestion rates of hTR_1–18_ RNA G4 sheds light on how different RNase digests RNA G4 structures (Figure 3H).

### 2.5 DHX36 Helicase Reduces the Fraction of Folded hTR_1–18_ G4s Thus Accelerates Their Degradation

**FIGURE 4:**
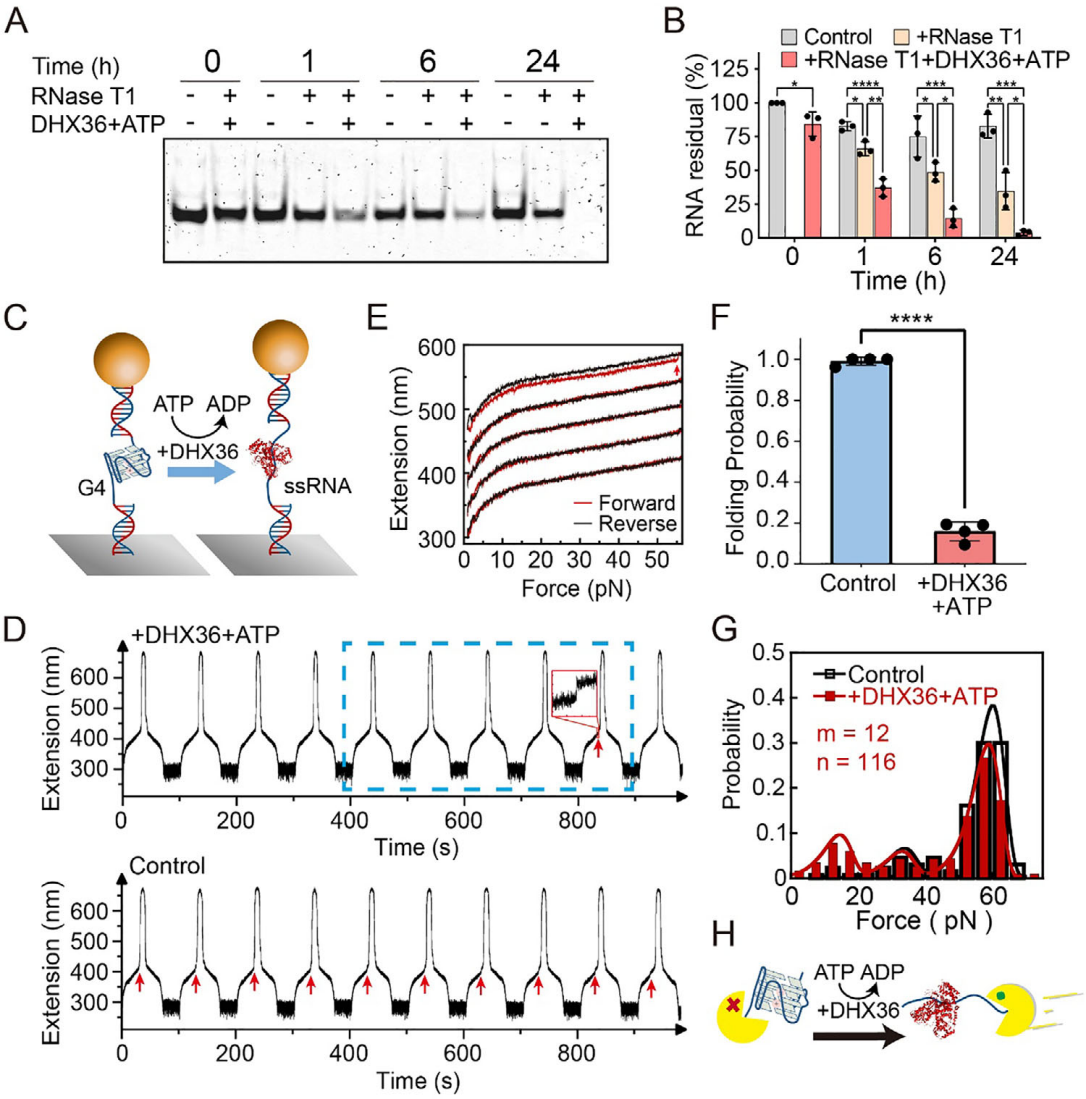
DHX36 reduces the folding probability of hTR‐15U RNA G4s and promotes their degradation. (A) Native PAGE analysis of hTR‐15U RNA G4s degradation accelerated by DHX36. hTR‐15U RNA G4s were treated under three conditions: G4s alone (control), G4s with RNase T1 (1 U/µL), and G4s plus RNaseT1 and DHX36 (5 nm) in the presence of ATP (1 mm). (B) Quantification of RNA band intensities from (A), normalized to the control sample at t = 0. Data are presented as average ± standard deviation from three independent experiments (*n* = 3). Statistical significance was determined by one‐way ANOVA with Tukey's post‐hoc test for multiple comparisons at each indicated time point (*p*‐values: *p < 0.05, **p < 0.01, ***p < 0.001, ****p < 0.0001). (C) Schematic illustration of the single‐molecule magnetic tweezers measurements. (D) Representative extension traces of hTR‐15U G4s with (upper panel) or without (lower panel) 5 nM DHX36 and 1 mm ATP in 100 mm KCl. (E) Overlay of representative stretching (red) and relaxing (black) extension curves from the blue dashed box in (D). For visual clarity, the five stretching curves are shifted 40 nm along the extension axis. The red arrows represent the G4 unfolding signal. (F) The folding probability of hTR‐15U G4s without or with DHX36 and ATP at a constant force holding time of 30 s. Statistical significance was determined by a *t*‐test. (G) The unfolding force histogram of hTR‐15U G4s without or with DHX36 and ATP. Data were compiled from n events across m molecules. (H) Schematic of DHX36's effect on the degradation of hTR‐15U G4s by RNase T1. The DHX36 structure is based on the coordinates from PDB ID 5N8U.

To investigate how the cell might regulate such an extraordinarily stable RNA G4 structure, we examined the effects of the G4‐specific RNA helicase DHX36. A 15 nt poly‐uracil (15U) single‐stranded tail was introduced at the 3′‐end of the G4‐forming sequence (hTR_1–18_) to facilitate the unwinding activity of DHX36 [51]. Hereafter, we named this construct hTR‐15U. The hTR‐15U RNA was pre‐folded in 10 mm Tris‐HCl (pH 8.0), 100 mm K^+^ buffer, and then incubated with 1 U/µL RNase T1 with or without DHX36 and ATP for various incubation times, and the products were analyzed by native PAGE (Figure 4A and Figure S11). In the absence of DHX36 and ATP, the hTR‐15U RNA displayed significant resistance to RNase T1, with 35 ± 14% of the RNA remaining intact even after 24 h (Figure 4B). In contrast, the addition of 5 nm DHX36 and 1 mm ATP dramatically accelerated degradation, leading to only 37% ± 7% of the hTR‐15U RNA remaining after just 1 h of RNase T1 digestion. After 24 h, only 4 ± 2% of the hTR‐15U RNA remained in the DHX36 and ATP group, confirming that DHX36 resolves the stable RNA G4 structure, thereby making the RNA susceptible to rapid cleavage by RNase T1. We also validated that hTR‐15U RNA G4s remained structurally stable in the presence of either 1 mm ATP alone or 5 nm DHX36 alone (Figures S12 and S13).

We next utilized single‐molecule magnetic tweezers to analyze the unwinding activity of DHX36 on hTR‐15U G4 structure (Figure 4C) [51]. The upper panel of Figure 4D shows the representative stretching cycles of hTR‐15U G4s in the presence of 5 nm DHX36 and 1 mm ATP. Each stretching step (at a loading rate of 2 pN/s) was followed by a relaxing step (at a loading rate of −2 pN/s) and a 1 pN hold for G4s refolding. G4 formation was detected by both the characteristic extension jump during stretching and by comparing the stretching and relaxing curves. Specifically, a higher extension in the relaxing curve than the stretching curve indicates that the RNA G4 was folded during the holding time and subsequently unfolded during stretching. In control experiments (without DHX36 and ATP), hTR‐15U G4 formation was observed in virtually all stretching cycles (Figure 4D lower panel). In contrast, in the presence of DHX36 and ATP, most stretching curves overlaid with the relaxing curves, indicating that the hTR‐15U remained in the unfolded ssRNA states (Figure 4D,E). By repeating this experiment across multiple molecules, we quantified this highly efficient unwinding by DHX36, as the G4s’ folding probability was reduced from near 99% ± 2% (117 cycles in 4 independent molecules) to 16% ± 5% (233 cycles in 4 independent molecules) (Figure 4F). This result demonstrates the efficient unwinding activity of DHX36, which strongly shifts the G4 folding equilibrium toward the unfolded ssRNA state.

The unfolding force distribution of hTR‐15U G4s in the presence of DHX36 and ATP was measured from the remaining 16% folded fraction (Figure 4F). This distribution revealed major unfolding force peaks (58 ± 4 pN (67%) and 39 ± 12 pN (13%)) that were similar to those observed in control experiments (major peak at 59 ± 5 pN (86%) and a minor peak at 37 ± 12 pN (14%)), both remains consistent with hTR_1–18_ RNA G4s (Figure 4G). In addition, a small fraction of events unfolded at a lower force of 13 ± 5 pN (20%), suggesting the presence of a potent intermediate or partially unwounded population during enzymatic unwinding. Taken together, these single‐molecule and RNase T1 digestion assays reveal that DHX36 efficiently unwound hTR_1–18_ RNA G4s in the presence of ATP, and renders the RNA susceptible to RNase degradation (Figure 4H).

## 3 Discussion

Using single‐molecule magnetic tweezers, we demonstrated that the RNA G4s formed at the 5'‐end of hTR (hTR_1–18_) possess exceptional kinetic stability (slow unfolding rate) at a physiologically relevant K^+^ concentration (100 mm). The predominant species of hTR_1–18_ RNA G4s exhibits a remarkably high unfolding force (peak at 58 ± 4 pN) and an extremely slow zero‐force unfolding rate (3.1 ± 0.3) × 10^−7^ s^−1^, corresponding to an intrinsic half‐life of a week. The profound resistance to RNase digestion is a direct functional consequence of this kinetic stability. Specifically, our data indicated that RNase T1 could potentially degrade the G4 structure with a slow digestion rate (*k*_dig_ = (1.2 ± 0.4) × 10^−5^ s^−1^), while the slower rate observed for RNase A (*k*_dig_ = (5 ± 2) × 10^−7^ s^−1^) suggesting that RNase A requires the G4s to be unfolded to access their target loop region. Quantifying RNA G4s’ unfolding rates is thus crucial for deciphering the precise molecular mechanism by which G4s confer resistance to RNase.

Our single‐molecule measurements reveal that the hTR_1–18_ RNA G4s possess a slower unfolding rates but faster folding rates compared to their DNA counterparts (Figures 1 and 2) and other parallel‐stranded DNA G4s [41]. It folds orders of magnitude faster and requires a significantly lower K^+^ concentration to form (*K*_0.5_ ≈ 0.03 mm for RNA vs ∼0.5 mm for DNA). This remarkable kinetic advantage ensures the rapid and efficient formation of the G4 scaffold immediately following hTR transcription. The observation enhanced the thermodynamic and kinetic stability of the RNA G4, likely conferred by the presence of extra 2’‐OH on the ribose sugar. Kinetically, the 2'‐OH group biases the ribose toward a C3'‐endo pucker and an anti‐glycosidic bond conformation [56]. This conformational pre‐organization streamlines the folding process into a rapid, funnel‐like pathway, as it avoids the slow syn/anti isomerization steps required for the folding of many DNA G4 topologies [31]. Thermodynamically, this pre‐organization reduces the entropic cost of folding. Moreover, the 2'‐OH groups introduce additional stabilizing interactions by forming an extensive hydrogen‐bonding network with the phosphate backbone and structured water molecules [27, 29]. Crucially, the stability of RNA G4s is also enhanced by synergistic effects between adjacent ribonucleotides and a more significant stabilizing contribution from the loop regions, which are less pronounced in their DNA counterparts [57]. This combination of a streamlined kinetic pathway and multiple, synergistic thermodynamic advantages collectively explains the exceptional stability of the hTR_1–18_ RNA G4s observed in our study.

The remarkable kinetic persistence of the hTR_1–18_ RNA G4s observed in vitro presents a challenge for cellular management. We addressed this by investigating how DHX36 helicase can overcome this extreme stability. Using the RNase T1 digestion assay, we showed that bovine DHX36 actively and efficiently resolves the hTR‐15U RNA G4 structures and renders it susceptible to RNase degradation. The high efficiency of DHX36 on the hTR‐15U RNA G4s was precisely quantified using our single‐molecule assay. This analysis showed that DHX36 achieves a level of unwinding efficiency (reducing the folded fraction from 99% to 16%) that is higher than previously reported for Drosophila DHX36, which destabilized parallel‐stranded DNA G4s primarily into less‐stable intermediate states (retaining 47% ± 9% of the folded fraction) [51]. Our quantitative data highlight the capacity of DHX36 to significantly shift the hTR‐15U RNA G4s equilibrium to the unfolded state.

The precise regulation of human telomerase is critical for genome stability. The extraordinary persistence of hTR_1–18_ RNA G4s strongly suggests that this structure functions as a durable structural hub. This long‐lived nature makes it suited to mediate functional processes, such as telomerase dimerization, and to provide a binding site for regulatory proteins, including DHX36 helicase, thereby ensuring the robust telomerase regulation [23, 26, 45]. Similarly, stable RNA G4s have been found to serve as transcription factor binding centers or promote the formation of stress granules [58, 59], thereby influencing a wide range of cellular processes.

The efficient resolution of the extraordinarily stable hTR_1–18_ G4s by DHX36 underscores the enzyme's critical role in regulating telomeric RNA G4 structure [23]. DHX36 processes a specific N‐terminal RHAU‐specific motif (RSM‐motif) and an OB‐fold domain that facilitates the recognition of G4s with high structural specificity [48, 60, 61]. Other helicase within the same DEAH‐box family, such as DHX9, which is also known to unfold G4 structures, bears an RGG‐box domain but lack the N‐terminal G4‐binding domain present in DHX36 [62]. Consequently, DHX9 functions as a more versatile helicase, frequently involved in unwinding dsRNA, dsDNA, and R‐loop [63]. Furthermore, helicases from different superfamilies, such as Pif1 and BLM, also resolve G4s but with distinct mechanistic features and topological preferences [64]. For example, Pif1 preferentially unwinds antiparallel G4s, while BLM employs context‐dependent mechanisms [64]. This illustrates that cells possess a toolkit of specialized helicases for G4 metabolism. Our findings firmly position DHX36 as the primary enzyme tailored to resolve highly stable parallel‐stranded RNA G4s.

## 4 Materials and Methods

### 4.1 RNA and DNA Constructs for Single‐Molecule Experiments

All oligonucleotides were custom‐synthesized by Sangon Biotech (China) or Genewiz (China). Two double‐stranded DNA (dsDNA) handles were prepared by two separate PCR amplifications using Lambda phage DNA (Thermo Fisher Scientific, USA) as the template. The primers for the two handles were labeled with a 5′‐biotin and 5′‐thiol, respectively, enabling specific surface attachments. The PCR products were digested with the restriction enzyme BstXI (Thermo Fisher Scientific, USA) and purified using a PCR purification kit (Tiangen Biotech, China). The central segment, an hTR_1–18_ RNA sequence flanked by two DNA sequences (termed hTR_1–18_‐MT, Table S1), was annealed with two complementary ssDNA sequences (Flank 1 and Flank 2). This module was then ligated to the dsDNA handles using T4 DNA ligase (Thermo Fisher Scientific, USA). To prevent RNA degradation during the ligation step, 0.2 U/µL RNAase inhibitor (Thermo Fisher Scientific, USA) was added. The final ligation product was analyzed by agarose gel electrophoresis to confirm the expected size and subsequently purified using a gel extraction kit (Tiangen Biotech Co., Ltd, China).

### 4.2 Single‐Molecule Magnetic Tweezer Experiments

The glass surface of the flow chamber was functionalized using a 1% (*v*/*v*) solution of (3‐Aminopropyl)triethoxy silane (APTES) (Cool Chemical Technology, China). The resulting amine‐modified slides were assembled with coverslips to form flow chambers (∼30 µL volume) as previously described [65]. The chamber was incubated with 1 mg/mL sulfosuccinimidyl 4‐(N‐maleimidomethyl)cyclohexane‐1‐carboxylate (Sulfo‐SMCC) (Hunan Hua Teng Pharmaceutical, China) for 30 min. After washing with 1 × PBS buffer, the nucleic acid constructs were added and incubated for 40 min. Subsequently, the chamber was filled with blocking buffer (1 × PBS (pH 7.4), 10 mg/mL bovine serum albumin (BSA) (Sigma–Aldrich, USA), 1 mm 2‐mercaptoethanol (Genview, USA), 10 µm ethylene diamine tetra acetic acid (EDTA) (Biosharp, China), 0.2 U/µL RNase inhibitor (Thermo Fisher Scientific, USA)) and incubated at 4°C for at least 4 h. This step also quenches remaining reactive maleimide groups. Streptavidin‐coated superparamagnetic Dynal M280 beads were then added and incubated for 10 min, enabling specific binding to the 5'‐biotin labeled end of tethered DNA. Finally, the working buffer (10 mm Tris‐HCl (pH 8.0),10 µm EDTA, 0.2 U/µL RNase inhibitor, and a specified concentration of KCl (ranging from 10 to 200 mm)) was added to the flow chamber. All buffers were prepared with DEPC‐treated water, and RNase inhibitor was added to a final concentration of 0.2 U/µL immediately before use to ensure RNA integrity. The force‐ramp experiments were carried out using BioPSI magnetic tweezers (BioPSI, Singapore) at a room temperature of 22°C–26°C.

### 4.3 Data Analysis for Single‐Molecule Magnetic Tweezer Experiments

The unfolding force distributions were analyzed using Bell's model,

(1) $$p\;\left(F\right)=\frac{k_{u}^{0}}{r}\;\exp\left\{\frac{\Delta x_{u}F}{k_{B}T}+\frac{k_{B}Tk_{u}^{0}}{\Delta x_{u}r}\left[1-\exp\left(\frac{\Delta x_{u}F}{k_{B}T}\right)\right]\right\}$$

where *r* is the force loading rate (2 pN/s), *k*_B_ is the Boltzmann constant, *T* is the absolute temperature, and Δ*x*_u_ is the distance to the transition state. For distributions exhibiting multiple peaks, the data were fitted to a linear combination of the corresponding single‐component functions. The overall probability density was modeled as *p*_total_(*F*) = Σ *α*_i_ * *p*_i_(*F*), where *α*_i_ is the fractional population of the i state, with the constraint that Σ *α*_i_ = 1. The number of nucleotides involved in the G4 structure was determined from the unfolding step sizes. The end‐to‐end distance *x* of the ssRNA segment released upon unfolding is the sum of the measured extension jump λ and the extension of the folded G4 structure *x*_G4_ (*x* = *λ* + *x*_G4_). The folded G4 was approximated as a rigid body with a size of $L_{0}^{G4}$ = 1 nm estimated from NMR structure. Its projected length along force direction, *x*_G4_(*F*), was calculated using the monomer force‐extension curve of the FJC model,

(2) $$x_{G4}\left(F\right)=L_{0}^{G4}\;\coth\left(\frac{FL_{0}^{G4}}{k_{B}T}\right)-\frac{k_{B}T}{FL_{0}^{G4}}$$

Subsequently, the contour length *L*_0_ of the released ssRNA was calculated from *x* at the unfolding force *F* using an extensible worm‐like chain (WLC) model [66]:

(3) $$L_{0}=x/\left(1-\frac{1}{2}\sqrt{\frac{k_{B}T}{FL_{p}}}+\frac{F}{K}\right)$$

For ssRNA, the persistence length *L*_p_ was set to 1.0 nm and the stretch modulus *K* to 1600 pN [67]. Finally, the number of nucleotides was calculated by dividing the counter length per nucleotide 0.59 nm/nt [67].

### 4.4 Circular Dichroism Spectroscopy and Melting Curve Measurements

Circular dichroism (CD) spectra were acquired using a Bio‐Logic MOS‐500 spectropolarimeter equipped with a temperature control unit. All measurements were conducted in a 10 mm path‐length quartz cuvette. For sample preparation, 4 µm hTR_1–18_‐CD or hTD‐CD oligonucleotides were dissolved in 10 mm Tris‐HCl buffer (pH 8.0) containing varying concentrations of KCl (0‐100 mM). Oligonucleotides were annealed by heating to 95°C for 5 min, followed by gradual cooling to room temperature. CD spectra were collected in the wavelength range of 220–320 nm, using a 1 nm step size, 0.5 s acquisition duration, and 2 nm bandwidth. The buffer contribution was subtracted from each sample spectrum, and data were zero corrected at 320 nm. Thermal melting was performed at several temperatures during heating from 25°C to 98°C at a rate of 3°C/min with a 1‐min equilibration time at each temperature. The melting temperatures (*T*_m_) were determined by fitting the melting curves (265 nm) to a sigmoid function. Thermodynamic parameters were determined by plotting ln *K*_eq_ (*T*) against 1/*T* (van't Hoff analysis). The enthalpy change (Δ*H*) and entropy change (Δ*S*) were determined from the slope (‐Δ*H*/R) and the y‐intercept (Δ*S*/*R*), respectively. The Gibbs free energy change (Δ*G*) was calculated using the equation Δ*G* = Δ*H‐T*Δ*S*.

### 4.5 Potassium Ion Titration Assay

The K^+^‐induced folding of the hTR_1–18_‐CD or hTD‐CD oligonucleotides was monitored by CD spectroscopy. Specified K^+^ concentrations were achieved by serial additions of concentrated KCl solution to a 4 µm oligonucleotide solution in 10 mm Tris‐HCl buffer (pH 8.0). After each addition, the solution was mixed thoroughly and incubated for 3 min to ensure equilibrium. CD spectra were then recorded from 220 to 320 nm on a Bio‐Logic MOS‐500 spectropolarimeter at room temperature. All spectra were baseline‐corrected by subtracting the spectrum of the corresponding buffer (10 mm Tris‐HCl, pH 8.0) containing the same KCl concentration to account for dilution and buffer effects. To quantify the K^+^‐dependence of G4 folding, the fraction of folded G4s at each point was determined from the change in molar ellipticity (Δ*θ*) at 265 nm, normalized to the total change observed upon saturation. The resulting titration curve was fitted to the Hill equation:

(4) $$\frac{\Delta \theta_{i}}{\Delta \theta_{f}}=\frac{{\left[K^{+}\right]}^{n_{H}}}{{K_{0.5}}^{n_{H}}+{\left[K^{+}\right]}^{n_{H}}}$$

where *K*_0.5_ is the K^+^ concentration required for half‐maximal folding and the Hill coefficient *n*_H_ reflects the cooperativity of K^+^ binding to G4s.

### 4.6 Stopped‐Flow Circular Dichroism Kinetics

Measurements were performed with a Bio‐Logic SFM‐4000 stopped‐flow system coupled to a CD spectropolarimeter. In each experiment, 10 µm pre‐annealed hTR_1–18_‐CD or hTD‐CD oligonucleotides in 20 mm Tris‐HCl buffer (pH 8.0) were rapidly mixed 1:1 (*v*/*v*) with KCl solution at a flow rate of 13.5 mL/s in a 1.5 mm path length cuvette. Final solutions after mixing contained 5 µm nucleic acid, 10 mm Tris‐HCl (pH 8.0), and KCl at concentrations of 1 or 100 mm. G4 folding kinetics were monitored by recording the change in ellipticity at 265 nm with a 5 nm bandwidth. Kinetic traces for hTR_1–18_ were acquired with a time resolution of 0.1 s, while a 0.5 s resolution was used for hTD. For most conditions, particularly for hTR_1–18_, the folding process was too rapid to be fitted, occurring within 0.1 s. However, for the slower‐folding hTD at 1 mm K^+^, a complete kinetic trace could be recorded and was well‐described by a single exponential function to extract the folding rate. For each K^+^ concentration, at least three independent kinetic traces were recorded. All measurements were performed at room temperature.

### 4.7 RNase T1 and RNase A Digestion Rate Measurement

4 µm hTR_1–18_‐Digestion oligonucleotides in 10 mm Tris‐HCl buffer (pH 8.0) were pre‐folded by annealing in the presence of specified concentrations of KCl. Subsequently, 1 µL RNase T1 (1000 U/µl, Biyotime Biotechnology, China) or 2 µL RNase A (10 mg/mL, Biyotime Biotechnology, China) was added to 1 mL RNA solution and immediately mixed. CD spectra were recorded from 220 to 320 nm at different time points throughout the course of the digestion to monitor the reaction progress. Digestion curves were generated by plotting the change in ellipticity at 265 nm against time and fitted to an exponential decay function θ(*t*) = θ_final_ *(1 + α*exp (− *k*_dig_*t)), where θ_final_ is the residual ellipticity after complete degradation, *k*_dig_ is the apparent degradation rate, and α is an amplitude coefficient related to the initial ellipticity. All experiments were performed at room temperature in triplicate.

### 4.8 Native PAGE Analysis of hTR‐15U RNA G4s Degradation with DHX36

The recombinant Bovine DHX36 protein was expressed in *Escherichia coli* and purified as previously described [48]. 3 µm hTR‐15U RNA oligonucleotides were folded by annealing in 10 mm Tris‐HCl (pH 8.0) and 100 mm KCl. Then the pre‐folded RNA was subjected to reaction under three parallel conditions: i) Helicase‐assisted digestion: The reaction was initiated by adding RNase T1 (1 U/µL final concentration), 5 nM DHX36, and an ATP regeneration system consisting of 1 mm ATP (Sigma–Aldrich, USA), 2 mm MgCl_2_, 2.5 mm phosphoenolpyruvate (Sigma–Aldrich, USA), and 200 µg/mL pyruvate kinase (Sigma–Aldrich, USA). ii) RNase‐only control: RNase T1 was added to a final concentration of 1 U/µL. iii) No‐enzyme control: An equivalent volume of nuclease‐free water was added instead of enzymes. Incubations were started 24, 6, and 1 h(s) before electrophoresis, respectively. The 0‐h sample was prepared immediately before loading. Following their respective incubation periods at room temperature, all samples were loaded onto a 15% native polyacrylamide gel in 0.5 × TBE buffer. The gel was stained with 4S Green Plus Nucleic Acid Stain (Sangon, China) for 30 min and analyzed using Amersham Typhoon RGB laser‐scanning imager (Cytiva, Japan).

### 4.9 Single‐Molecule Unwinding Assay of hTR G4 by DHX36

The hTR‐15U constructs were prepared and assembled in the flow chamber following the procedures described above. The mechanical properties of hTR‐15U G4s were characterized in a working buffer with 100 mm KCl, 10 mm Tris‐HCl (pH 8.0), 10 µm EDTA, and 0.2 U/µL RNase inhibitor. The unfolding force distribution and refolding probability were determined from repeated force‐ramp cycles, using a 30 s holding time at 1 pN for the folding. Subsequently, the buffer was carefully exchanged for a reaction buffer containing 5 nm DHX36, 1 mm ATP, 2 mm MgCl_2_, and an ATP regeneration system (2.5 mm phosphoenolpyruvate, 200 µg/mL pyruvate kinase) diluted in working buffer.

### 4.10 RNase‐Free Protocol

To minimize the risk of RNase contamination, stringent measures were implemented. Pipettes and the working area were cleaned with RNase decontaminant (Huayueyang Biotechnology, China) before sample preparation. Enzyme‐free pipette tips and centrifuge tubes were used exclusively. Buffers in contact with RNA were prepared using DEPC‐treated water (Sangon Biotech, China) and were renewed at least every 10 days. All RNA manipulations, including the preparation of single‐molecule magnetic tweezers samples, were conducted within an RNA‐specific operational area.

### 4.11 Statistical Analysis

The identification of unfolding events by analyzing raw force‐extension curves was performed with LabVIEW (NI, USA) and MATLAB (MathWorks, USA). For single‐molecule force spectroscopy, n represents the total number of unfolding events collected from m individual molecules. Specific n and m values for each experiment are provided in the respective figure. Data from bulk biochemical assays (Circular Dichroism, Native PAGE) are presented as mean ± standard deviation (SD) from at least three independent experiments. Statistical significance between experimental groups in the Native PAGE assays was determined by one‐way ANOVA with Tukey's post‐hoc test for multiple comparisons using Prism 9.0 (GraphPad Software, USA). A *p*‐value < 0.05 was considered statistically significant (*p < 0.05, **p < 0.01, ***p < 0.001, ****p < 0.0001).

## Funding

This work was financially supported by the National Natural Science Foundation of China (32571415, 32171225).

## Conflicts of Interest

The authors declare no conflicts of interest.

## Supporting information




**Supporting File**: advs74430‐sup‐0001‐SuppMat.pdf.

## Data Availability

The data that support the findings of this study are available in the supplementary material of this article.
