# Supplementary material for: Extreme Kinetic Stability and RNase Resistance of Human Telomerase RNA G‐Quadruplexes Overcome by DHX36 Helicase
Source: Adv Sci (Weinh). 2026 Feb 15;13(24):e22779. doi: 10.1002/advs.202522779 (PMC13116126; doi:10.1002/advs.202522779)
Supplement: Supplementary file 1 — Supporting File: advs74430‐sup‐0001‐SuppMat.pdf. [file ADVS-13-e22779-s001.pdf]

Supporting Information

Extreme Kinetic Stability and RNase Resistance of Human Telomerase RNA G-Quadruplexes Overcome by DHX36 Helicase

Qun Luo[1,†], Yashuo Zhang[1,2,†], Yingxian Qian[1], Huiying Zhan[1], Wenqiang Wu[2], and Huijuan You[1,]*

Q. Luo, Y. Zhang, Y. Qian, H. Zhan, H. You

Hubei Key Laboratory of Natural Medicinal Chemistry and Resource Evaluation, School of Pharmacy, Tongji Medical College, Huazhong University of Science and Technology, Wuhan, China

E-mail: youhuijuan@hust.edu.cn

Y. Zhang

Department of Pharmacy, Ruijin Hospital, Shanghai Jiao Tong University School of Medicine, Shanghai, China

W. Wu

State Key Laboratory of Crop Stress Adaptation and Improvement, Academy for Advanced Interdisciplinary Studies, School of Life Sciences, Henan University, Kaifeng, China

Table of Contents

Supplementary Tables

Table S1. Sequences of oligonucleotides used in this work.

Table S2. Thermodynamic parameters for hTR_{1-18} G4s and hTD G4s.

Supplementary Figures

Figure S1. Preparation scheme for the hTR_{1-18} construct for magnetic tweezer experiments.

Figure S2. Representative extension traces of hTR_{1-18} RNA G4s during force-ramp cycles.

Figure S3. Representative 32 consecutive force-ramp cycles from a single hTR_{1-18} molecule

Figure S4. Unfolding force and step size distribution of hTR-nobulge G4s.

Figure S5. CD spectrum and unfolding step size distribution of hTD G4s.

Figure S6. K^+ titration CD spectra of hTR_{1-18} (A) and hTD (B) titrated with a range of K^+ concentrations.

Figure S7. Representative CD spectra recorded during thermal denaturation experiments of hTR_{1-18} in buffers with different K^+ concentrations.

Figure S8. Thermal analysis of the hTD DNA G4s.

Figure S9. A representative kinetic trace monitoring the digestion of hTR_{1-18} by RNase T1 (1 U/μL) in a K^+-free buffer.

Figure S10. Time-resolved CD spectra of hTR_{1-18} digested by RNase T1 (1 U/μL) at three different KCl concentrations.

Figure S11. Representative replicates of Native PAGE analysis of hTR-15U RNA G4s degradation.

Figure S12. Structural integrity of hTR-15U RNA G4s is maintained in the presence of 1 mM ATP.

Figure S13. Structural integrity of hTR-15U RNA G4s is maintained in the presence of DHX36.

Supplementary Tables

Table S1. Sequences of oligonucleotides used in this work

Name	Sequence (5' to 3')
$hTR_{1\text{-}18}$-MT	CTTGTGCACAGACTCGTArGrGrGrUrUrGrCrGrGrArGrGrGrUrGrGrGrCTGCAGCC AGGTCAGTAGCGAC
hTR-nobulge-MT	CTTGTGCACAGACTCGTArGrGrGrUrUrGrGrGrArGrGrGrUrGrGrGrCTGCAGCCA GGTCAGTAGCGAC
hTD-MT	CTTGTGCACAGACTCGTAGGGTTGCGGAGGGTGGGTTGCAGCCAGGTCAGTA GCGAC
$hTR_{1\text{-}18}$-CD	TArGrGrGrUrUrGrCrGrGrArGrGrGrUrGrGrGrCT
hTD-CD	TAGGGTTGCGGAGGGTGGGTT
hTR-nobulge-CD	TArGrGrGrUrUrGrGrGrArGrGrGrUrGrGrGrCT
$hTR_{1\text{-}18}$-Digestion	rArGrGrGrUrUrGrCrGrGrArGrGrGrUrGrGrGrC
hTR-15U	rArGrGrGrUrUrGrCrGrGrArGrGrGrUrGrGrGrCrUrUrUrUrUrUrUrUrUrUrUrUrUrUrU
hTR-15U-MT	CTTGTGCACAGACTCGTArArGrGrGrUrUrGrCrGrGrArGrGrGrUrGrGrGrCrUrUrUr UrUrUrUrUrUrUrUrUrUrUrUTGCAGCCAGGTCAGTAGCGAC
Handle 1-FW	ATCACCAAGTGCATGGTGCTTGAACCCGCCTATG
Handle 1-RV	Thiol-CGACTGAGCTGGCAAGCAACTGACCTG
Handle 2-FW	ATCACCAACGACATGGCAGGAGGGCGAATG
Handle 2-RV	Biotin-CGAAGCAGATCCCACGCAACCAGCTTACGG
Flank 1	Phosphate-CTACTGACCTGGCTGC
Flank 2	CGAGTCTGTGCACAAGGTGC

RNA is indicated in red, DNA in black.

Table S2. Thermodynamic parameters for $hTR_{1\text{-}18}$ G4s and hTD G4s

Name	[KCl] (mM)	T_m (°C)	ΔH (kcal·mol^{-1})	ΔS (cal·K^{-1}·mol^{-1})	ΔG_{25} (kcal·mol^{-1})
$hTR_{1\text{-}18}$ RNA G4s	100	87.1 ± 0.5	-62 ± 6	-174 ± 16	-10.4 ± 0.9
	50	84.4 ± 0.4	-64 ± 9	-180 ± 25	-10 ± 2
	20	79.7 ± 0.1	-66 ± 3	-187 ± 7	-10.0 ± 0.4
	10	76.8 ± 0.1	-61 ± 1	-174 ± 4	-8.8 ± 0.2
	1	64.6 ± 0.2	-58 ± 4	-173 ± 11	6.6 ± 0.4
	0	44.2 ± 0.4	-41 ± 6	-131 ± 19	-2.3 ± 0.3
hTD DNA G4s	100	64.6 ± 0.1	-48 ± 1	-141 ± 4	-5.5 ± 0.2

Supplementary Figures

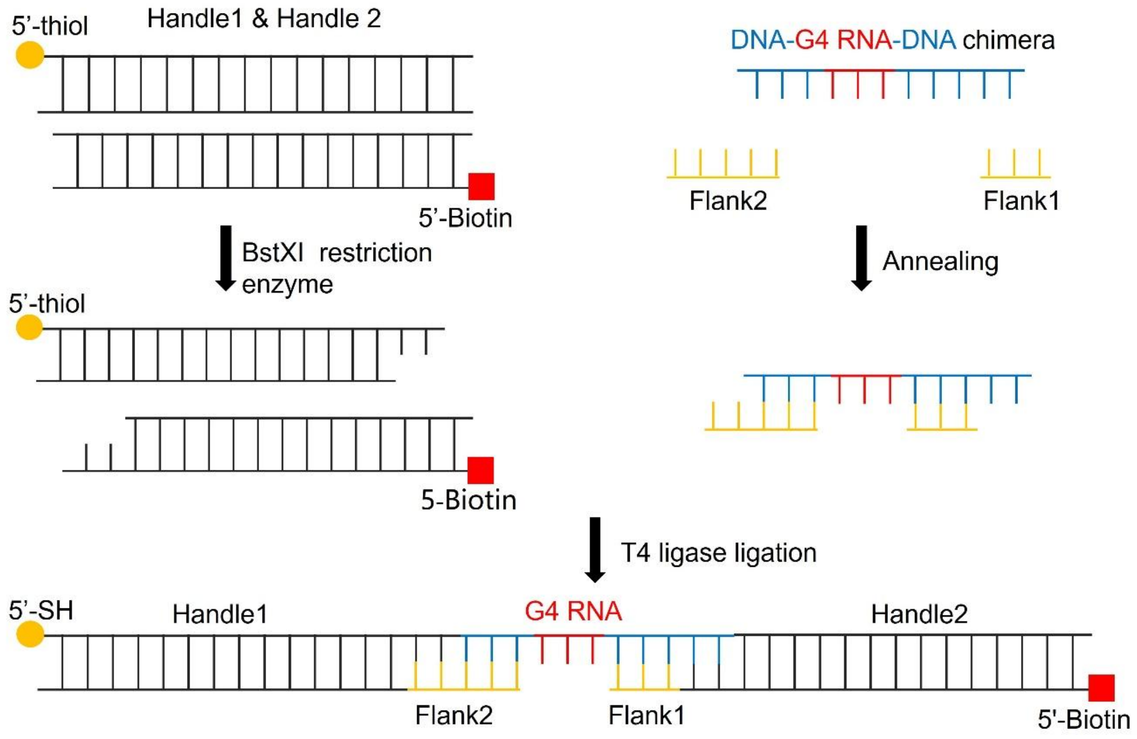

Figure S1. Preparation scheme for the hTR_{1-18} construct for magnetic tweezer experiments. The left panel shows the preparation of two dsDNA handles. Handle 1 was PCR amplified using a 5'-thiol labeled primer (for attachment to the coverslip), while Handle 2 was PCR amplified using a 5'-biotin labeled primer (for attachment to the magnetic bead). Both handles were digested with BstXI to generate cohesive ends for ligation. The right panel shows the center segment, the hTR_{1-18}-MT sequence (a chimeric oligonucleotide containing the hTR_{1-18} G4-forming region), which was annealed with two complementary ssDNA strands (Flank 1 and Flank 2) to prepare the central ligation module. Finally, the two handles and the central ligation module were subsequently ligated using T4 DNA ligase to create the final tether. Both dsDNA handles have high GC content (>60%) to prevent DNA melting when DNA is held at high forces.

The complete sequence of Handle 1 obtained after PCR amplification is:
ATCACCAAGTGCATGGTGCTTGAACCCGCCTATGCGCGGGTTTTCTTTTGTGCGCTTGCAGGCCAGCTTGG
GATCAGCAGCCTGACGGATGCGGTGTCCGGCGACAGCCTGACTGCCCAGGAGGCACTCGCGACGCTGGC
ATTATCCGGTGATGATGACGGACCACGACAGGCCCGCAGTTATCAGGTCATGAACGGCATCGCCGTGCTGC
CGGTGTCCGGCACGCTGGTCAGCCGGACGCGGGCGCTGCAGCCGTACTCGGGGATGACCGGTTACAACG
GCATTATCGCCCGTCTGCAACAGGCTGCCAGCGATCCGATGGTGGACGGCATTCTGCTCGATATGGACACG
CCCGGCGGGATGGTGGCGGGGGCATTTGACTGCGCTGACATCATCGCCCGTGTGCGTGACATAAAACCGG
TATGGGCGCTTGCCAACGACATGAACTGCAGTGCAGGTCAGTTGCTTGCCAGCTCAGTCG

The complete sequence of Handle 2 obtained after PCR amplification is:
ATCACCAACGACATGGCAGGAGGGCGAATGACCAAAGAGACTCAATCAACAACTGTTTCAGCCACTGCTTC
GCAGGCTGACGTTACTGACGTGGTGCCAGCGACGGAGGGCGAGAACGCCAGCGCGGCGCAGCCGGACGT

GAACGCGCAGATCACCGCAGCGGTTGCGGCAGAAAACAGCCGCATTATGGGGATCCTCAACTGTGAGGAG
GCTCACGGACGCGAAGAACAGGCACGCGTGCTGGCAGAAACCCCCGGTATGACCGTGAAAACGGCCCGCC
GCATTCTGGCCGCAGCACCACAGAGTGCACAGGCGCGCAGTGACACTGCGCTGGATCGTCTGATGCAGGG
GGCACCGGCACCGCTGGCTGCAGGTAACCCGGCATCTGATGCCGTTAACGATTTGCTGAACACACCAGTGT
AAGGGATGTTTATGACGAGCAAAGAAACCTTTACCCATTACCAGCCGCAGGGCAACAGTGACCCGGCTCAT
ACCGCAACCGCGCCCGGCGGATTGAGTGCGAAAGCGCCTGCAATGACCCCGCTGATGCTGGACACCTCCA
GCCGTAAGCTGGTTGCGTGGGATCTGCTTCG

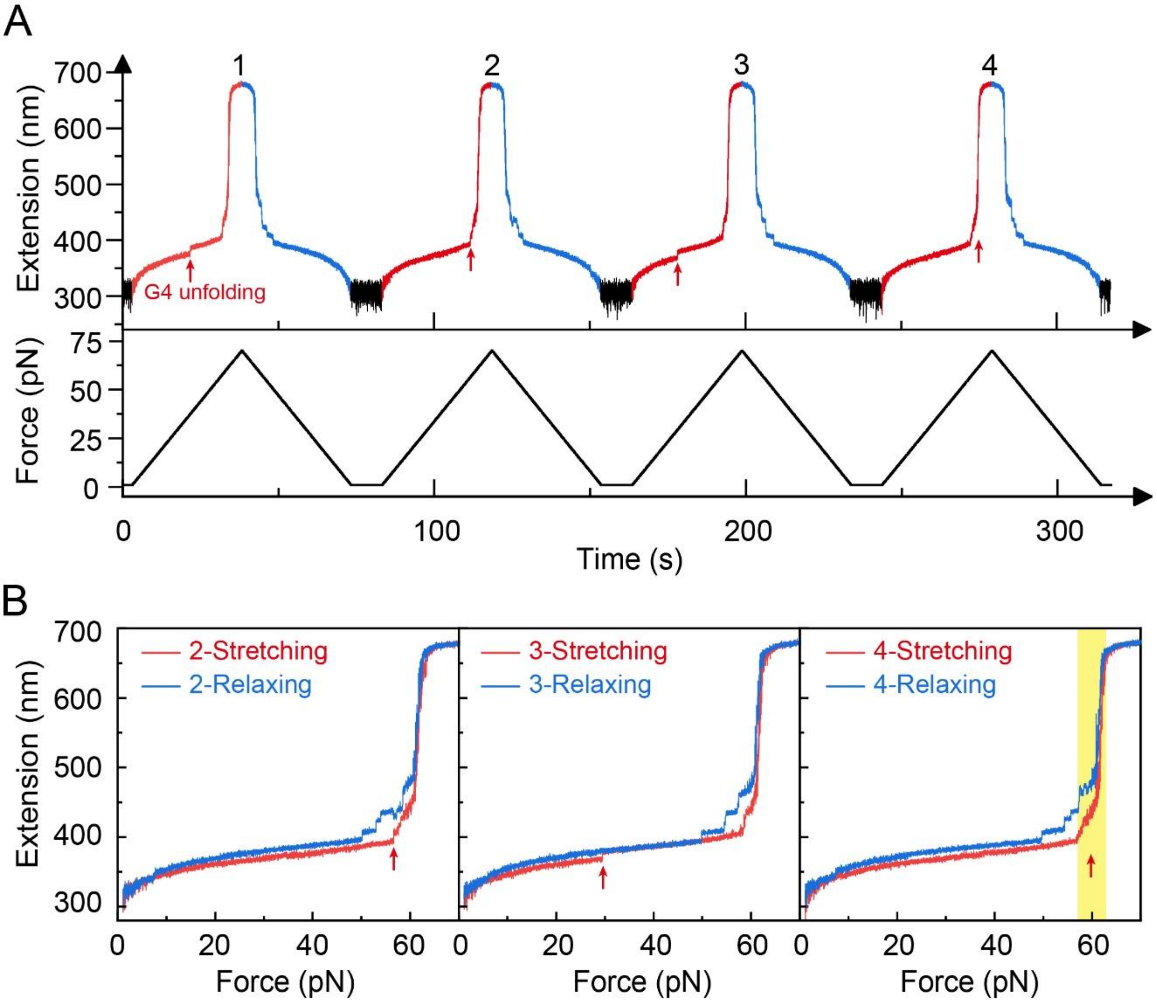

Figure S2. Representative extension traces of hTR1-18 RNA G4s during the force-ramp cycles. (A) Representative force-ramp cycles. A hTR1-18 molecule was subjected to repeated stretching (1 to 70 pN) and relaxing cycles (70 to 1 pN) at a constant rate of 2 pN/s. The cycle number is indicated above the trace. The red arrows indicate the G4 unfolding events. (B) Comparison of the stretching (red) and relaxing (blue) force-extension curves. Individual stretching (red) and relaxing (blue) force-extension curves are overlaid for direct comparison. The higher extension of the relaxing curve than the stretching curve suggests G4 formation in cycles 2, 3 and 4. For cycle 4, the G4 unfolding event falls within the B-to-S overstretching transition regime. The mid-point value of the transition was used to calculate the G4s unfolding force.

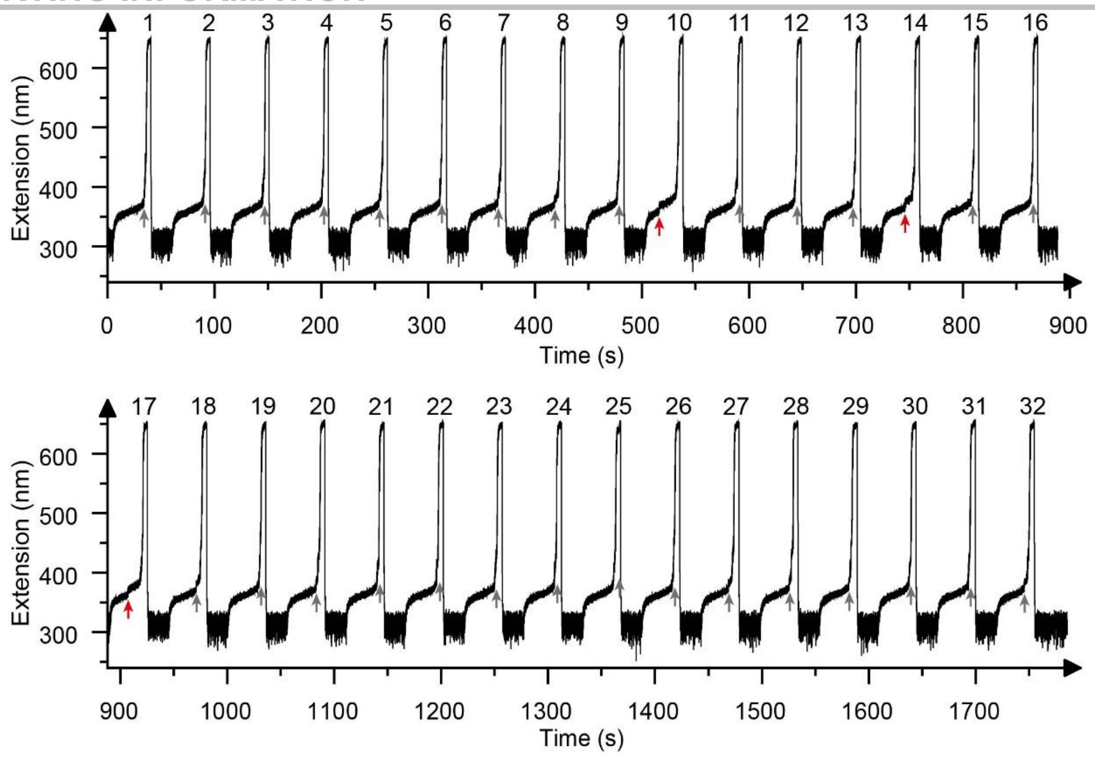

Figure S3. Representative 32 consecutive force-ramp cycles from a single hTR_{1-18} molecule. The vast majority of unfolding events occur at high forces (gray arrows). However, rare unfolding events at significantly lower forces (< 45 pN) are clearly observed (red arrows, e.g., in cycles 10, 14, 17), demonstrating that the same molecule can access the less stable conformation.

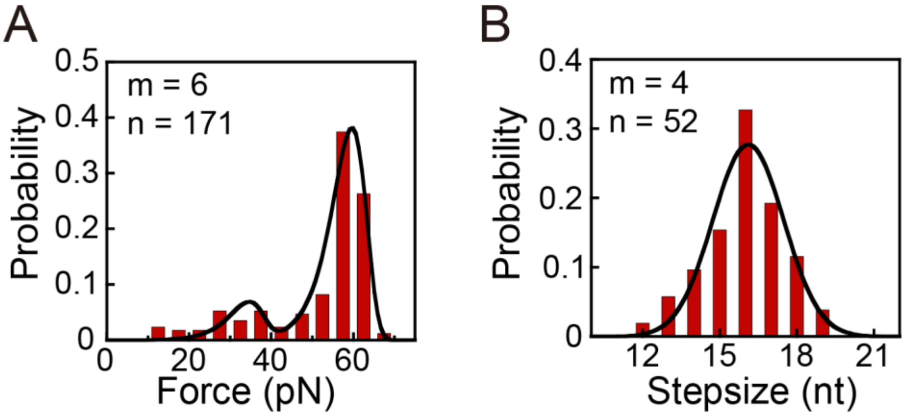

Figure S4. Unfolding force and step size distributions of hTR-nobulge G4s. (A) The unfolding force distribution of hTR-nobulge in 100 mM KCl showed a minor population at 34 ± 7 pN (average ± standard deviation) and a major population at 59 ± 4 pN. The solid line is a fit to a two-component Bell's model, which yielded zero-force unfolding rates k_u^0 of (1.1 ± 0.6) × 10^{-4} s^{-1} (average ± fitting error) for the low-force state (15% of events) and (2.4 ± 0.2) × 10^{-7} s^{-1} for the high-force state (85% of events). (B) The distribution of unfolding step sizes is centered at 16 ± 1 nt (average ± standard deviation), consistent with the expected length of the hTR-nobulge G4-forming sequence. The solid line is a fit to a Gaussian function. Data were compiled from n events across m molecules.

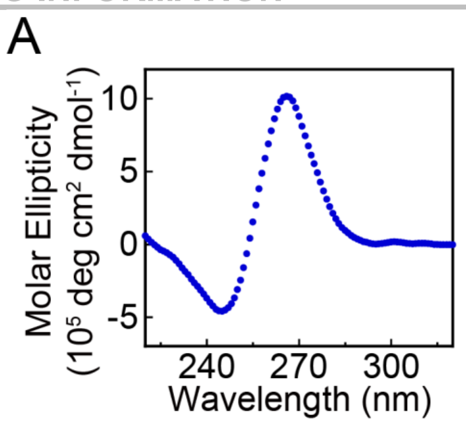

Figure S5. CD spectrum and unfolding step size distribution of hTD G4s. (A) The CD spectrum of hTD in 100 mM K^+ exhibits a positive maximum at ~265 nm and a negative minimum at ~245 nm, the canonical signature of a parallel-stranded G4 topology. (B) The unfolding step size distribution of hTD is centered at 17 ± 1 nt (average ± standard deviation), which matches the number of nucleotides in the hTD G4-forming sequence.

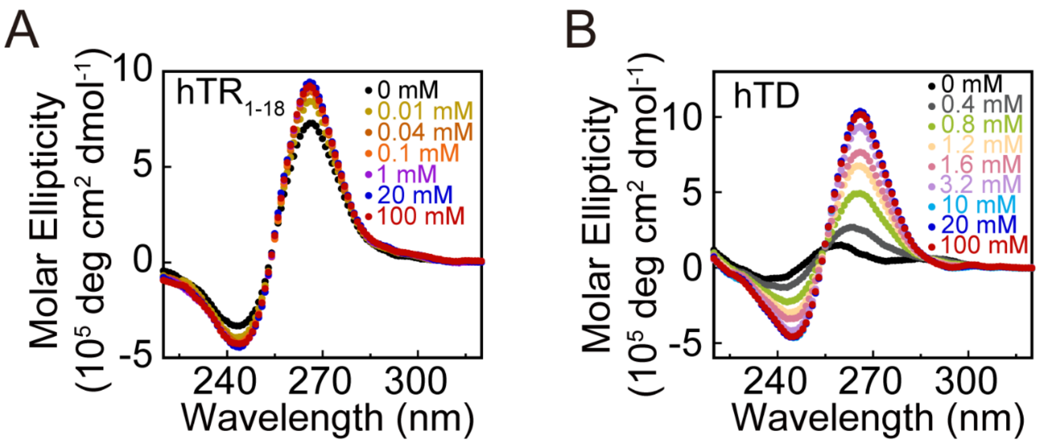

Figure S6. K^+ titration CD spectra of $hTR_{1\text{-}18}$ (A) and hTD (B) titrated with a range of K^+ concentrations. (A) The $hTR_{1\text{-}18}$ RNA G4s adopt the canonical parallel topology (positive peak at ~265 nm) even in a K^+-free buffer (0 mM, black). (B) The hTD DNA analogue is predominantly unstructured in the absence of K^+, and the gradual titration of KCl is required to induce the conformational transition to the parallel G4 structure, which only approaches saturation at millimolar concentrations. All spectra were recorded at room temperature.

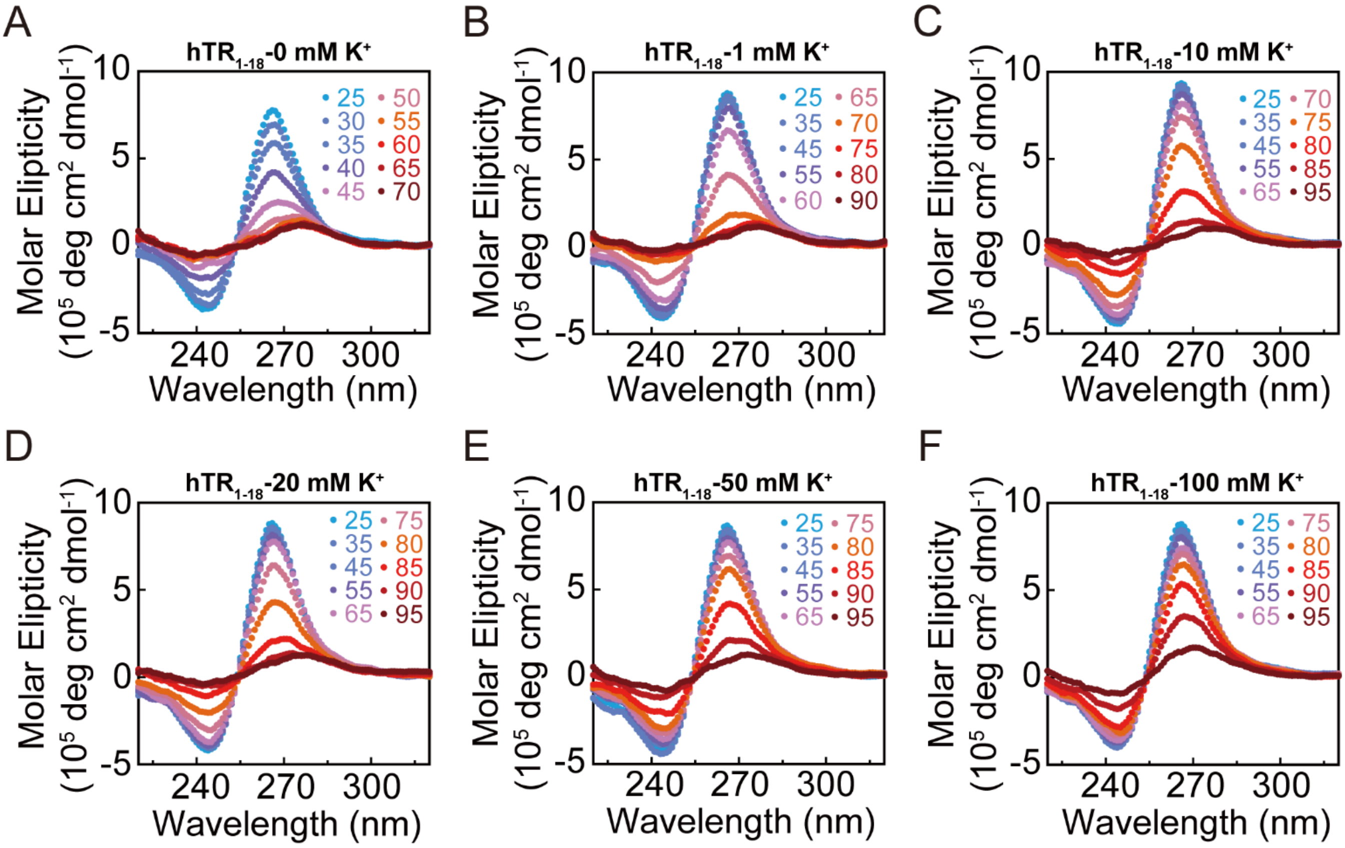

Figure S7. Representative CD spectra recorded during thermal denaturation experiments of hTR1-18 in buffers with different K^+ concentrations. The progressive decrease in the characteristic parallel G4 signature at ~265 nm upon heating (indicated by the color gradient from blue to red) reflects the unfolding of the structure.

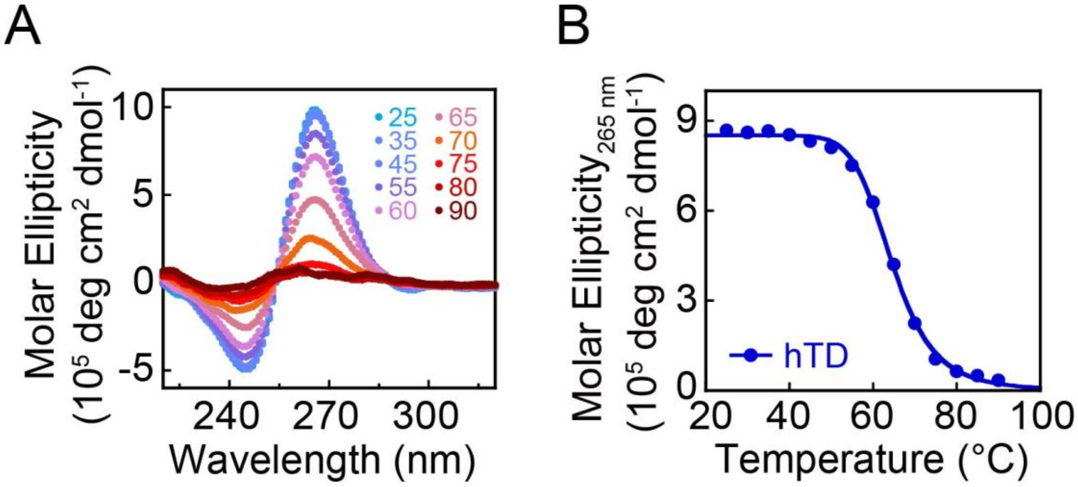

Figure S8. Thermal analysis of the hTD DNA G4s. (A) Representative temperature-dependent CD spectra of hTD G4s in 100 mM KCl. (B) Representative thermal melting curve, generated by plotting the ellipticity at 265 nm against temperature. The solid line is a fit to a sigmoidal function, which yields a melting temperature (T_m) of 64.6 ± 0.1°C (average ± standard deviation from three replicates).

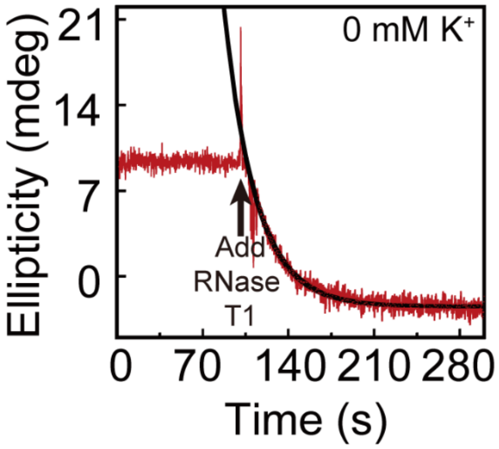

Figure S9. A representative kinetic trace monitoring the digestion of hTR$_{1-18}$ by RNase T1 (1 U/µL) in a K^+-free buffer. The decay of the CD signal at 265 nm indicates the rapid loss of residual structure upon enzymatic cleavage. The solid black line is a fit to a single-exponential decay model, which yields an apparent degradation rate constant (k_{dig}) of 0.05 ± 0.01 s^{-1} (average ± standard deviation from three replicates).

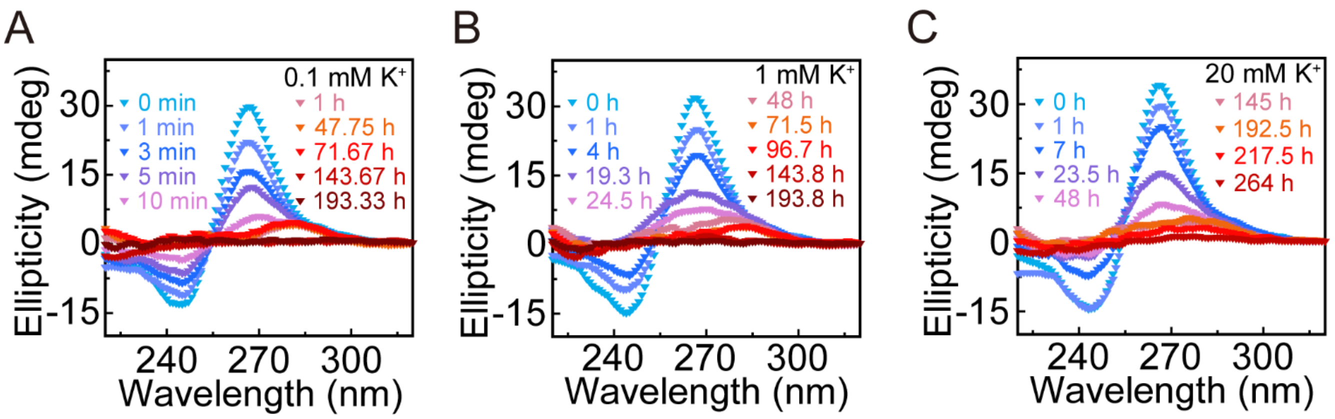

Figure S10. Time-resolved CD spectra of hTR$_{1-18}$ digested by RNase T1 (1 U/µL) at three different KCl concentrations: (A) 0.1 mM, (B) 1 mM, and (C) 20 mM.

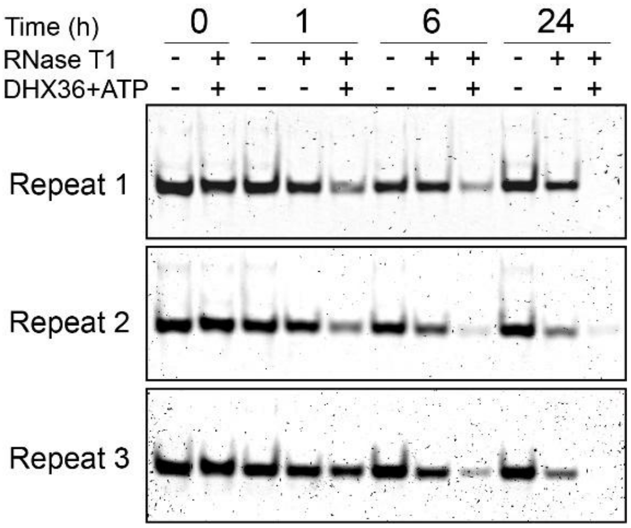

Figure S11. Representative replicates of Native PAGE analysis of hTR-15U RNA G4s degradation. This figure displays three independent biological replicates of the RNaseT1 digestion assay. The quantitative statistical analysis derived from these replicates is presented in Figure 4B of the main text.

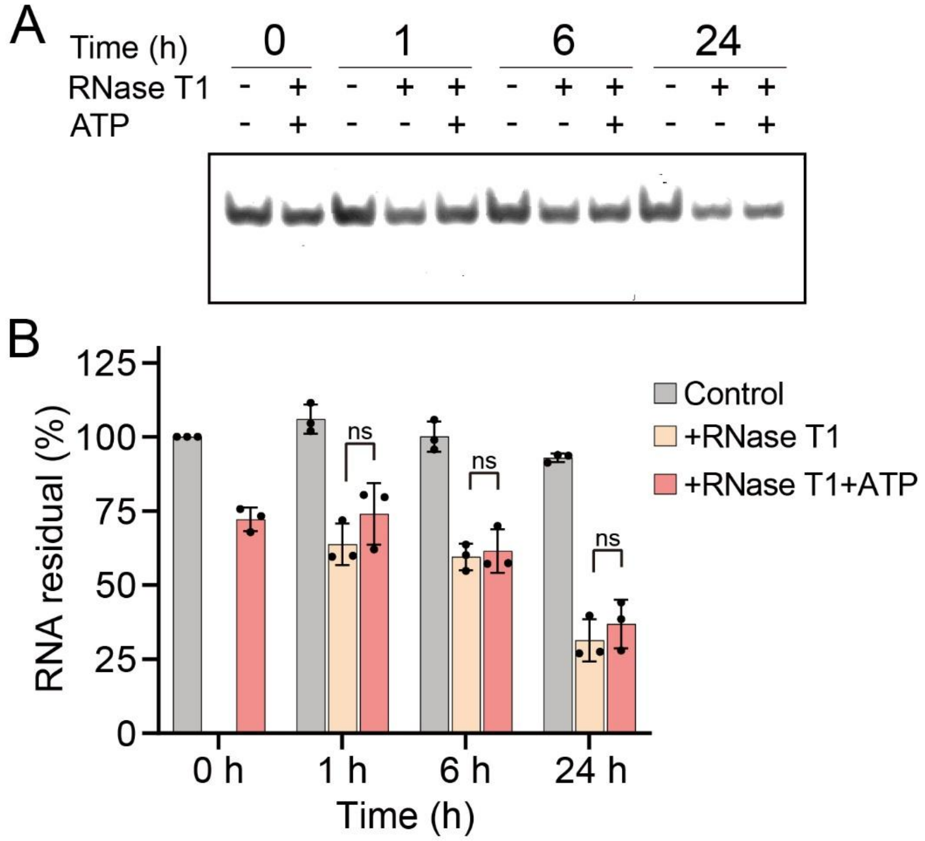

Figure S12. Structural integrity of hTR-15U RNA G4s is maintained in the presence of 1 mM ATP. (A) Representative Native PAGE analysis of hTR-15U RNA G4s degradation. hTR-15U RNA was incubated under three conditions: G4s alone (control), with RNase T1 (1 U/μL), and with both RNase T1 (1 U/μL) and ATP (1 mM). Samples were collected at the indicated time points 0, 1, 6, and 24 hours. (B) Quantification of relative RNA band intensities from (A), normalized to the control sample at t = 0. Data are presented as average ±

standard deviation from three independent experiments (n = 3). Statistical significance was determined by one-way ANOVA with Tukey's post-hoc test for multiple comparisons at each indicated time point (ns, not significant, *p < 0.05, **p < 0.01, ***p < 0.001, ****p < 0.0001).

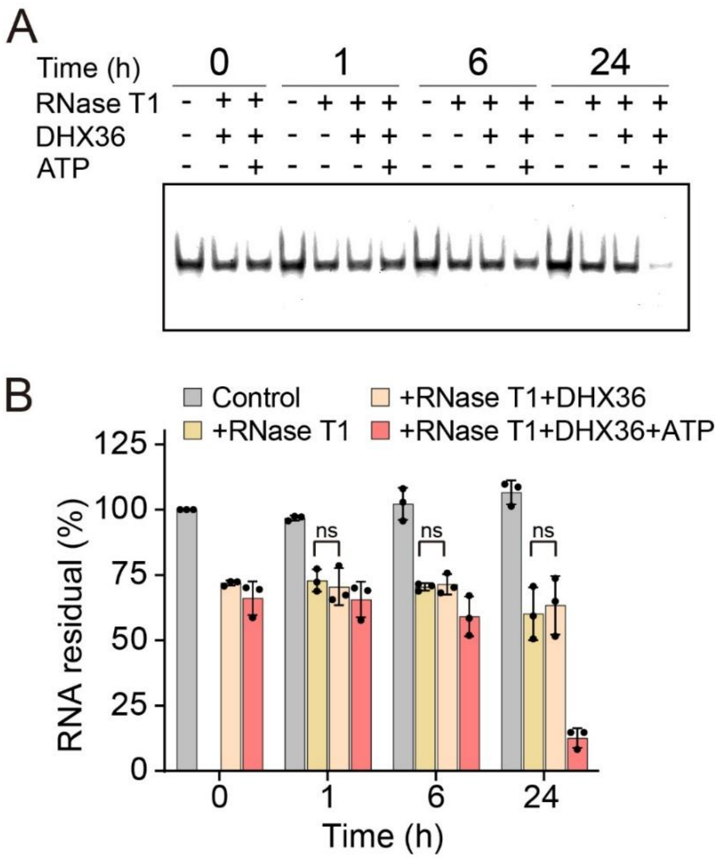

Figure S13. Structural integrity of hTR-15U RNA G4s is maintained in the presence of only DHX36. (A) Representative Native PAGE analysis of hTR-15U RNA G4s degradation. hTR-15U RNA (5-10 μM) was incubated under three conditions: G4s alone (control), with RNase T1 (1 U/μL), with both RNase T1 (1 U/μL) and DHX36 (5 nM), and with RNase T1 (1 U/μL), DHX36 (5 nM), and 1 mM ATP. Samples were collected at the indicated time points 0, 1, 6, and 24 hours. (B) Quantification of relative RNA band intensities from (A), normalized to the control sample at t = 0. Data are presented as average ± standard deviation from three independent experiments (n = 3). Statistical significance was determined by one-way ANOVA with Tukey's post-hoc test for multiple comparisons at each indicated time point (ns, not significant, *p < 0.05, **p < 0.01, ***p < 0.001, ****p < 0.0001).
